# Support for a radiation of free-living flatworms in the African Great Lakes region and the description of five new *Macrostomum* species

**DOI:** 10.1186/s12983-023-00509-9

**Published:** 2023-09-05

**Authors:** Jeremias N. Brand

**Affiliations:** 1 Department of Environmental Sciences, Zoological Institute, University of Basel, Vesalgasse 1, Basel, 4051 Switzerland; 2 Department of Tissue Dynamics and Regeneration, Max Planck Institute for Multidisciplinary Science, Am Fassberg 11, 37077 Göttingen, Germany

## Abstract

**Background:**

The African Great Lakes have long been recognized as an excellent location to study speciation. Most famously, cichlid fishes have radiated in Lake Tanganyika and subsequently spread into Lake Malawi and Lake Victoria, where they again radiated. Other taxa have diversified in these lakes, such as catfish, ostracods, gastropods, and Monegenean gill parasites of cichlids. However, these radiations have received less attention, and the process leading to their speciation in this unique region remains to be further explored. Here I present evidence that suggests a radiation of *Macrostomum* flatworms has occurred in the African Great Lakes region, offering a good opportunity for such investigations.

**Results:**

Recent field work has revealed a monophyletic clade of 16 *Macrostomum* flatworms that have, to date, only been collected from Lake Tanganyika. Additionally, a species collected from Lake Malawi was found nested within this clade. Molecular phylogenetic analysis, largely based on transcriptome data, suggests that this clade underwent rapid speciation, possibly due to a large habitat diversity in the lake. I also observed significant differences in the sperm morphology of these flatworms compared to those of species found outside Lake Tanganyika and Lake Malawi. These included the elongation of an anterior structure, a reduction in the size of the lateral sperm bristles, and changes in relative proportions. I propose functional hypotheses for these changes in sperm design, and formally describe *Macrostomum gracilistylum* sp. nov from Lake Malawi and its sister species *Macrostomum crassum* sp. nov., *Macrostomum pellitum* sp. nov., *Macrostomum longispermatum* sp. nov., and *Macrostomum schareri* sp. nov., from Lake Tanganyika.

**Conclusions:**

The available evidence is consistent with the hypothesis that *Macrostomum* flatworms have radiated in Lake Tanganyika and subsequently spread to Lake Malawi. However, whether this represents a *bona fide* adaptive radiation still needs to be determined. Therefore, the African Great Lakes are promising targets for further research into flatworm diversity and speciation.

**Supplementary Information:**

The online version contains supplementary material available at 10.1186/s12983-023-00509-9.

## Introduction

Adaptive radiations give rise to extraordinary species diversity, diversity of morphology, and ecological specialization. Radiations can occur when organisms arrive in a new habitat such as islands, as in the well know radiations of Darwin finches on the Galapagos [1], Anole lizards in the Caribbean [2, 3], or fruit flies on the Hawaiian island chain [4]. Similarly, lakes can serve as substrates of radiations, like in the famous diversification of cichlid fishes in the African Great Lakes [5, 6]. These radiations are characterized by speciation coupled with diversification and adaptation to new ecological niches [7]. Numerous investigations have delved into the impact of habitat availability on the development and pace of radiation [8]. Additionally, studies centered on adaptive radiations have uncovered that sexual selection can play a crucial role in radiations. Sexual selection can pre-partition species via reproductive barriers (e.g., via allopatric divergence and subsequent reproductive isolation upon secondary contact [9]) or it can maintain linkage disequilibrium between adaptive traits through assortative mating [10].

The definitions of what a radiation entails are diverse [8], but a widely used definition is the one proposed by [7]: An adaptive radiation must satisfy common ancestry, a phenotype-environment correlation, trait utility, and rapid speciation. However, since collecting evidence for each of these criteria requires considerable effort, not all studied systems have support for all these criteria [8]. Therefore, it is helpful to identify promising study systems using a broader definition, such as: ‘the rapid […] origin of taxonomic, ecological and morphological diversity as a consequence of adaptation to novel or hitherto underutilized ecological niches‘ [7, 11, 12].

Here I report on a promising study system recently discovered in a global sampling campaign of free-living flatworms in the genus *Macrostomum* [13]. These small (1–3 mm body length) flatworms occur in aquatic habitats across a very broad salinity spectrum [14]. They are typically benthic, living in interstitial spaces between sand grains (meiofauna) in lakes, streams, and ponds, but some species live on various types of water plants and other submerged substrates.

**Fig. 1:**
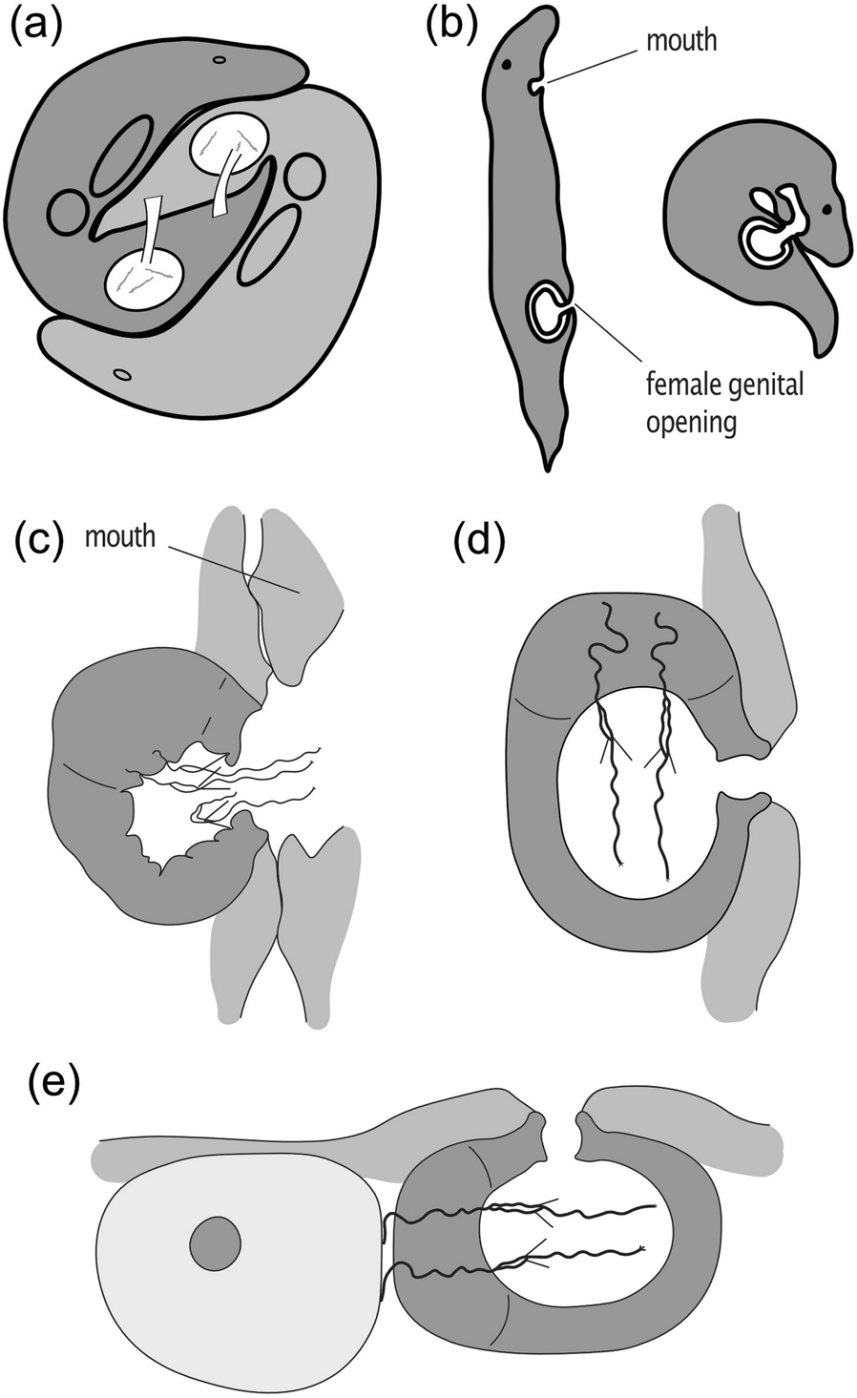
Functional hypotheses for the changes in sperm design observed in the Tanganyika clade. **a** Typical mating posture during reciprocally mating *Macrostomum* species. **b** Anatomical details during the suck behavior. **c** During the suck behavior, both sperm feelers and bristles are hypothesized to anchor sperm and prevent its removal. **d** and **e** Sperm with an elongated feeler or an extended anterior length could penetrate deep inside the recipient’s tissue and lead to stronger anchoring. **d** and/or allow egg-sperm contact (**e**). Images (**a**–**c**) are redrawn, and (**d**–**e**) are modified from [21]

**Fig. 2:**
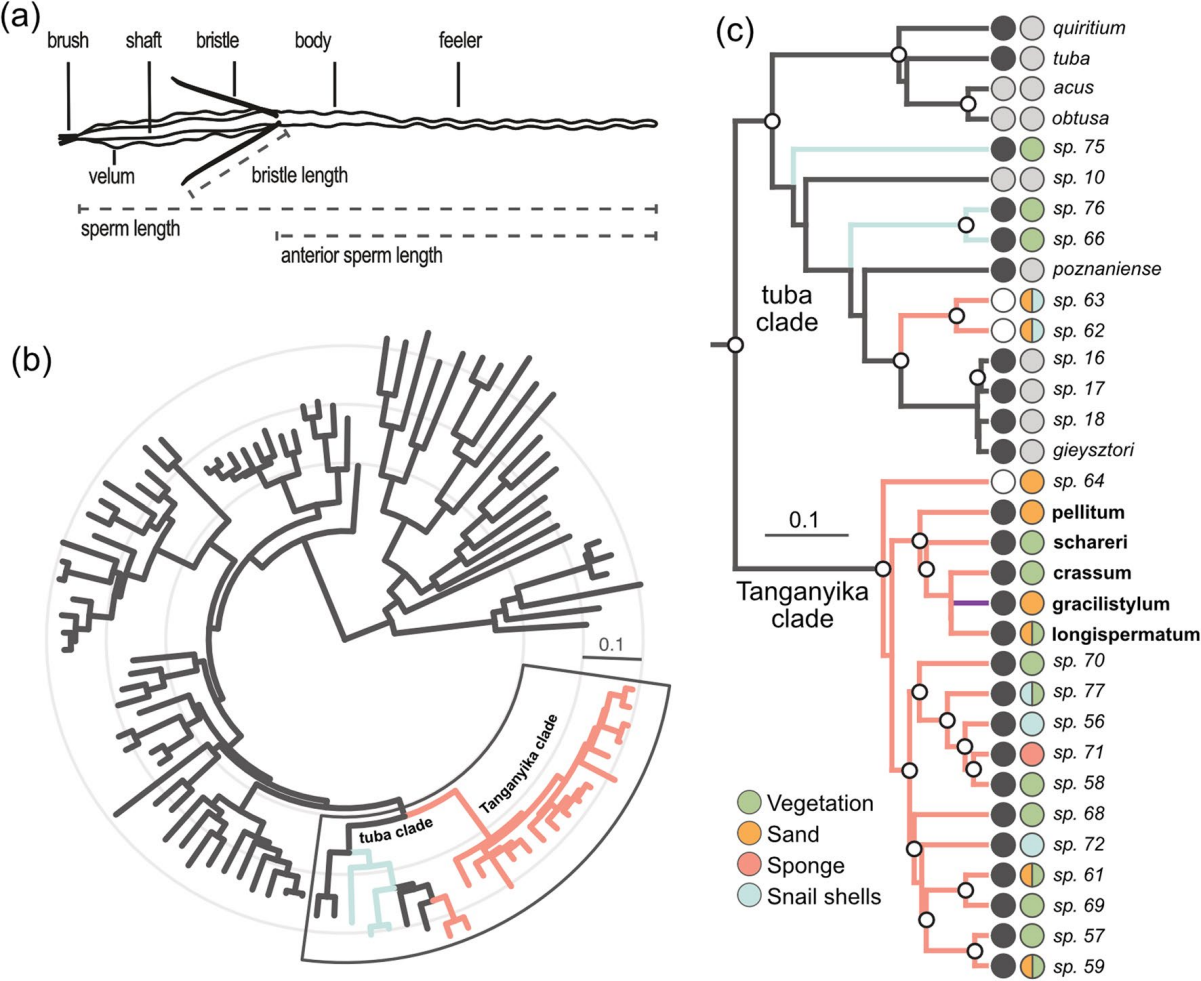
Detail on sperm morphology and phylogenetic trees. **a** *Macrostomum* sperm schematic with key structures and measured traits. **b** Phylogeny (H-IQ-TREE) of 96 *Macrostomum* species from [13], based on the alignment of 94,625 amino acids across 385 genes. Concentric circles: 0.1 substitutions/site. Branch colors: collection locations (red: Lake Tanganyika, blue: Zambia, black: other). Note the short branches at the base of the Tanganyika, indicating rapid speciation. **c** Phylogeny (C-IQ-TREE) of the outlined clades from (**b**), based on the alignment from (**b**) supplemented with sequences from a *28 S rRNA* fragment to include species without transcriptome data, namely *M. acus*, *M. obtusa*, *M. gracilistylum*, *M.* sp. 10, *M.* sp. 16, *M.* sp. 17, and *M.* sp. 18. Note that because only one gene is present for these species their branch-lengths are not as suitable for the assessment of the diversification rate. White circled nodes: maximum support. First column circles: mating syndrome from [19] (black: reciprocal, white: hypodermic, grey: not assigned). Second column circles: habitat type (see legend). Dual-color circles: species from multiple habitats. Branch colors, as in (**b**)

The Genus *Macrostomum* is known for its diverse reproductive morphology and mating behavior [15–19]. These species are simultaneous hermaphrodites, and many of them engage in reciprocal copulation, where both partners can receive sperm during a single mating interaction (Fig. 1a). After copulation, many of these reciprocally mating species exhibit the so-called “suck behavior” [20], in which the worms apparently try to remove the received ejaculate (Fig. 1b) [21]. The sperm of these reciprocally mating species often have stiff lateral bristles (Fig. 2a), which likely help anchor the sperm in the female reproductive tract preventing its removal during the suck behavior (Fig. 1c) [21]. The anterior sperm region may also help anchor sperm during the suck behavior (Fig. 1c). However, across the genus, the sperm bristles have repeatedly been lost or reduced, and these losses are correlated with the evolution of traumatic mating via hypodermic insemination [19] and are linked to genome-wide changes in selection pressures [22, 23]. During hypodermic insemination, a worm injects sperm through the partner’s epidermis using a needle-like copulatory organ, and the injected sperm then moves through the tissue to the site of fertilization. In summary, the Genus *Macrostomum* exhibits a wide range of reproductive strategies, including reciprocal copulation and traumatic insemination, and the presence or absence of sperm bristles is evolutionarily linked to these different mating behaviors [15, 19].

*Macrostomum* is a diverse and interesting study system for examining the macroevolutionary effects of sexual selection and sexual conflict [19, 24]. This is especially true because it includes the model organism *M*. *lignano* Ladurner, Schärer, Salvenmoser, & Rieger, 2005 [25, 26], which has been used to study pre- and post-copulatory sexual conflict [15, 27–29] and sex allocation [30, 31]. Finally, genomic resources have been developed for the entire genus, including multiple high-quality transcriptome assemblies [32–34] and a molecular phylogeny encompassing 145 species [13].

Here I discuss the distribution of *Macrostomum* species collected in Lake Tanganyika, Lake Malawi, and surrounding waterbodies. I highlight a species flock in Lake Tanganyika, which I call the Tanganyika clade. Based on a comparative analysis of sperm traits, I describe a novel trait in the Tanganyika clade, and I discuss hypotheses regarding its function. Next, I formally describe five species of the Tanganyika clade that represent the morphological diversity across the clade and have an interesting geographic distribution. Of these five *Macrostomum* species, four were from the southern tip of Lake Tanganyika, Zambia, namely, *Macrostomum crassum* Brand, sp. nov., *Macrostomum pellitum* Brand, sp. nov., *Macrostomum longispermatum* Brand, sp. nov., and *Macrostomum schareri* Brand, sp. nov., and one species from the southern region of Lake Malawi, Malawi, namely *Macrostomum gracilistylum* Brand, sp. nov. Moving on to the broader implications of these findings, I argue that the Tanganyika clade could constitute an adaptive radiation in the broad sense of the term.

## Methods

### Sample collection

I extensively documented the specimens for the species description as previously described [15, 25, 35], using primarily published digital photomicrographs [13]. Light microscopes (Olympus BH2) equipped with digital cameras (Ximea xiQ MQ042CG-CM) and differential interference contrast (DIC) were used to capture images and videos at various magnifications (ranging from 40× to 1000×). To observe the sperm, the tail of a worm was amputated just anterior to the seminal vesicle, and the seminal vesicle was subsequently ruptured using a cover slip (as described in [36]). The released sperm were documented using DIC and, on occasion, phase-contrast microscopy. The cut tail was then often preserved by replacing the mounting medium with lactophenol and sealing the slide with VALAP (1:1:1 mixture of petroleum jelly, lanolin, and paraffin). These whole-mount permanent preparations of the stylet were deposited at the Biodiversity Museum Göttingen, Germany. All documented specimens were assigned an ID in the MTP LS XXXX format, to which reference is made throughout the manuscript. Most specimen documentation and all molecular data have previously been deposited (Table 1, [13]). Detailed images and videos of all specimens are available as zenodo archives with the following DOIs: 10.5281/zenodo.2602479, 10.5281/zenodo.5656981, and 10.5281/zenodo.6353143.

**Table 1:** Information on the specimens analyzed of five *Macrostomum* species, *M. crassum*, *M. pellitum*, *M. longispermatum*, *M. schareri*, and *M. gracilistylum*

Species	Lake	Sample	Latitude, Longitude	ID	28 S	RNA-Seq	Whole mount
*M. crassum*	Tanganyika	J1-2	− 8.62291, 31.20062	**MTP LS 2644**	–	–	ZMUG 30,551
		B1	− 8.62291, 31.20062	MTP LS 2521	MT428735	SAMN15061070^(1)^	–
*M. pellitum*	Tanganyika	H8	− 8.61589, 31.19784	**MTP LS 2600**	–	–	ZMUG 30,552
				MTP LS 2585	MT428745	SAMN15061076^(1)^	–
				MTP LS 2586	–	–	ZMUG 30,553
				MTP LS 2587	–	–	–
				MTP LS 2588	–	–	–
				MTP LS 2615	–	–	ZMUG 30,554
				MTP LS 2616	–	–	ZMUG 30,555
				MTP LS 2617	–	–	–
				MTP LS 2623	–	–	–
		P3-4	− 8.61589, 31.19784	MTP LS 2730	–	–	–
				MTP LS 2733	–	–	–
				MTP LS 2736	MT428746	–	–
				MTP LS 2737	–	–	–
				MTP LS 2738	–	–	–
				MTP LS 2739	–	–	–
				MTP LS 2740	–	–	–
		F7	− 8.65532, 31.19380	MTP LS 2565	–	–	ZMUG 30,556
				MTP LS 2566	–	–	ZMUG 30,557
				MTP LS 2568	–	–	–
*M. longispermatum*	Tanganyika	H8	− 8.61589, 31.19784	**MTP LS 2598**	–	–	ZMUG 30,558
				MTP LS 2584	–	–	–
				MTP LS 2599	–	–	ZMUG 30,559
				MTP LS 2605	–	–	–
				MTP LS 2612	MT428751	SAMN15061079^(1)^	–
				MTP LS 2613	–	–	ZMUG 30,560
				MTP LS 2624	–	–	ZMUG 30,561
				MTP LS 2681	–	–	–
				MTP LS 2683	–	–	–
				MTP LS 2684	–	–	–
		K6	− 8.60285, 31.18399	MTP LS 2626	–	–	ZMUG 30,562
		O1	− 8.62257, 31.20022	MTP LS 2695	–	–	–
*M. schareri*	Tanganyika	M1	− 8.62257, 31.20022	**MTP LS 2688**	–	–	ZMUG 30,563
				MTP LS 2685	–	–	–
				MTP LS 2687	–	–	–
				MTP LS 2690	–	–	–
				MTP LS 2691	MT428761	SAMN15061083^(1)^	–
		O1	− 8.62257, 31.20022	MTP LS 2697	–	–	ZMUG 30,564
				MTP LS 2698	–	–	–
				MTP LS 2699	–	–	–
				MTP LS 2700	–	–	–
				MTP LS 2707	–	–	–
				MTP LS 2708	–	–	–
				MTP LS 2709	–	–	ZMUG 30,565
				MTP LS 2710	–	–	ZMUG 30,566
				MTP LS 2711	–	–	–
				MTP LS 2712	–	–	–
				MTP LS 2713	–	–	–
				MTP LS 2714	–	–	–
				MTP LS 2724	–	–	–
*M. gracilistylum*	Malawi	A1–2	− 14.28047, 35.10779	**MTP LS 3547**	––	––	ZMUG 30,567
				MTP LS 3545	–	–	–
				MTP LS 3546	–	–	ZMUG 30,568
				MTP LS 3548	–	–	ZMUG 30,569
				MTP LS 3549	–	–	ZMUG 30,570
				MTP LS 3550	–	–	ZMUG 30,571
				MTP LS 3551	MT428885	–	–
				MTP LS 3552	MT428886	–	–
				MTP LS 3554	–	–	ZMUG 30,572

Details are given on the location coordinates of the collection site, internal MTP LS collection ID (bold if HOLOTYPE, underlined if PARATYPE), the NCBI accessions for partial *28 S rRNA* sequences, the SRA accession for whole-body RNA-Seq data, and the voucher IDs for the deposited whole-mount preparations at the Biodiversity Museum Göttingen, Germany. (1) The transcriptomes assembled based on these data are deposited at: https://zenodo.org/record/4543289

### Morphometrics

Morphometric measurements were performed on digital images, or frames extracted from video recordings, in ImageJ software (version 1.53c, [37]) with the ObjectJ plugin (version 1.05j, https://sils.fnwi.uva.nl/bcb/objectj) to mark images non-destructively. Measurements were calibrated using a stage micrometer. Body length was measured by a segmented line along the central body axis. Body width was measured by a line perpendicular to the body axis at the height of the testes. Testis and ovary lengths were defined as the longest distance of the structure parallel to the body axis. Eye diameter, sensory cilia length, rhabdite granule length, sperm bristle length, and sperm brush length were measured by placing a straight line along the structures. Segmented stylet length was measured as the average length of segmented lines along both sides of the stylet. The definition of straight stylet length and the width of the stylet opening varied between species and is indicated in the respective line drawings. Total sperm length was measured as a segmented line from the most distal part of the feeler to just the anterior of the brush, as described in [19]. Some previous research has measured sperm feeler length (e.g., [36]). However, I did not consider this measurement as it was impossible to distinguish accurately the boundary between the sperm body and the feeler in the species presented here. Instead, I measured anterior sperm length, defined as the part of the total sperm length line anterior to the insertion point of the sperm bristles (Fig. 2a). As a consequence of this definition, it is not possible to measure anterior sperm length in species without bristles. Note that data on sperm bristle length, total sperm length, and segmented stylet length were taken from previous analyses of these specimens [19].

### Habitat type

To get an indication of the ecological niche the species occupy, I categorized the habitats in which the specimens were collected in Zambia and Malawi into four types. I distinguished sand (specimens from sand without any objects larger than 5 mm), snail shells(specimens from snail shell beds, including sand immediately surrounding it), sponges (specimens directly extracted from sponge tissue), and vegetation (specimens extracted from any water plant sample). Note that vegetation includes various substrates, including roots of reeds, various types of water plants, and pieces of rotting wood. In general, these types are broad classifications since our sampling was not aimed at detailed habitat characterization (and such data is often not available for *Macrostomum* species).

### Image processing

The images presented in the species description panels were processed using Photoshop (version: 21.2.1) to enhance image brightness and contrast with the “Brightness/Contrast” or “Levels” function and to crop and rotate as appropriate. Because some sperm extended across several images, I manually stitched them. Finally, I used the “Photomerge” function to generate a composite image of the stylet of two species derived from a video focusing through the structure. Note that such images can mask curvature in the z-axis.

### Comparative analysis

Since the newly described species and most members of the Tanganyika clade appeared to have an unusual sperm morphology, I compared four sperm traits across the genus. I selected only species with sperm bristles since this allowed analysis of a complete dataset. Furthermore, I included only species that were classified as reciprocally mating as opposed to hypodermically inseminating or intermediate mating since I wanted to compare species with the same general mating behavior. Following these criteria, I collected data on the total sperm and bristle lengths of 64 *Macrostomum* species from previous work [19]. Additionally, for this study, I measured the anterior sperm length and then calculated the sperm ratio as the species mean values of anterior sperm length over total sperm length (Fig. 2a). See Additional file 1: Table S1–2, for measurements for each specimen, species averages, and sample sizes.

I then performed a phylogenetically corrected comparative analysis to determine if the phylogenetic distribution of sperm traits supports a shift in trait evolution in the Tanganyika clade. I used a transcriptome-based maximum likelihood phylogeny (called C-IQ-TREE in [13]) generated based on the concatenated alignment of 385 proteins (94,625 amino acid positions) and a *28 S rRNA* fragment (787 nucleotide positions). The *28 S rRNA* fragment was used to integrate species without transcriptomic data. I mapped the total sperm length, sperm ratio, and bristle length onto the phylogeny of the genus and fit a multi-Ornstein-Uhlenbeck model of trait evolution using the R package *l1ou* (v1.43, [38]). Ornstein-Uhlenbeck models describe a random walk of traits along the branches of the phylogeny centered around an attractive optimum [39]. *l1ou* uses a LASSO approach to test if traits share a common optimum across the phylogeny or if shifts in these optima have occurred. Support for a shift in optima indicates that a statistical model with a new optimum on the given branch is a better fit for the data. In *l1ou*, such an inference can be applied to multiple traits simultaneously, and I applied it to all three traits, presented as the main model. I used a random state at the root and selected the best shift configuration model using the corrected Akaike information criterion. I then performed 100 non-parametric bootstraps of the best shift configuration. Additionally, I performed several analyses to evaluate the robustness of the results. I examined all other shift configurations that differed from the chosen configuration by less than 10 ΔAICc. I also ran the model with each trait individually, again examining several shift configurations. Finally, since the analysis was restricted to species for which I could measure all traits, I also analyzed the total sperm length (a trait that can be measured in all species), including a larger set of 92 species.

I tested if the Tanganyika clade had significantly different trait values compared to all other species using phylogenetically corrected generalized least-squares (PGLS) with the lambda model implemented in the R-package phylolm (v.2.6.2).

### Electron microscopy

Specimens of *M. schareri* were relaxed for several minutes using 0.1% 2-Phenoxypropanol in habitat water and then fixed in Trump’s Phosphate with 4% Paraformaldehyde and 1% Glutaraldehyde and stored at 4 °C. Samples were prepared for scanning electron microscopy (SEM) imaging following a modified protocol [40]. The most significant modification was a longer incubation time with the Durcupan resin resulting in a more thorough infiltration of the sample (A detailed protocol is included in Additional File 2). Fixed and stained samples were cut into rectangular blocks and glued to aluminum stubs using cyanoacrylate glue. Samples were trimmed to a block face for serial block-face SEM (SBFSEM) imaging with a microtome. Images were acquired using a variable pressure SEM (QuantaFEG 200; FEI) at low vacuum (~ 0.3 Torr) to alleviate issues with the charging of the sample. The microscope was equipped with an automated ultramicrotome inside the vacuum chamber (3View, Gatan) that was controlled by DigitalMicrograph software (Gatan). Backscattered electrons were detected by a silicon diode detector (Opto Diode Corp., USA) and preamplified by the preamplifier of the 3View system (Gatan). Specimens were sectioned along the anterior-posterior axis with a section thickness of 200 nm and a typical pixel size of 17 × 17  nm^2^ (range 17–27 nm^2^). The resulting image stacks were aligned along the x- and y-axis using the ImageJ plugin TrakEM2. TrakEM2 was also used to perform 3D reconstruction of the stylet by semi-automatic labeling of the sclerotized structure in every section. The resulting model was then exported, and a smoothed surface was calculated using the smooth function in Meshmixer (v3.5).

### Species delimitation

I followed the same species delimitation as previously described [13]. Briefly, I delimited species that showed at least four mutational differences in a sequenced *28 S rRNA* fragment or diagnostic differences in morphology.

## Results

### Geographic distribution and phylogenetics relationships

Since common descent and rapid speciation are key characteristics of an adaptive radiation, we examined previously published phylogenetic data for these characteristics. These analyses have revealed a *Macrostomum* clade containing one species collected from Lake Malawi (previously named *M*. sp. 117 and described in the present study as *M. gracilistylum*) and 16 species collected from Lake Tanganyika (hereafter called “Tanganyika clade”). The Tanganyika clade was monophyletic in all molecular analyses in Brand et al. [13] with maximal node support. Based on transcriptome data (available for all species except for *M. gracilistylum*), all species in the Tanganyika clade are distinct (Fig. 2c). However, note that, although morphologically distinct, some species differed in only one position in the haplotype network of a 787 nt *28 S rRNA* fragment (Fig. 22A in the supporting information of [13]), indicating recent divergence. Additionally, the internal branches within the Tanganyika clade are short, suggesting rapid speciation (Fig. 2b). In summary, these data show that the Tanganyika clade is monophyletic and had diverged rapidly.

However, while the Tanganyika clade was monophyletic, two species (*M*. sp. 62 and *M*. sp. 63) also collected from Lake Tanganyika did not cluster with the clade but instead grouped with a consistently recovered sister clade (referred to as the “tuba clade”, Fig. 2c [13]). Furthermore, the tuba clade contained three species (*M*. sp. 66, *M*. sp. 75, and *M*. sp. 76), which were collected from rivers and lakes in Zambia but not from Lake Tanganyika. The sample coordinates are described in the supporting information of Brand et al. [13].

### Habitat diversity

Examination of the habitats from which species were collected revealed diverse habitat types within Lake Tanganyika. I found eleven species on vegetation, eight in the sand, five in snail shells, and one species in a sponge (Fig. 2c). In contrast, the three species collected outside Lake Tanganyika or Lake Malawi (*M*. sp. 66, *M*. sp. 75, and *M*. sp. 76) were all found in vegetation. Notably, habitat types were not phylogenetically clustered in the Tanganyika clade (e.g., the snail shell habitat was likely colonized several times independently, Fig. 2c), consistent with the diversification of species into newly available ecological niches.

### Sperm evolution

**Fig. 3:**
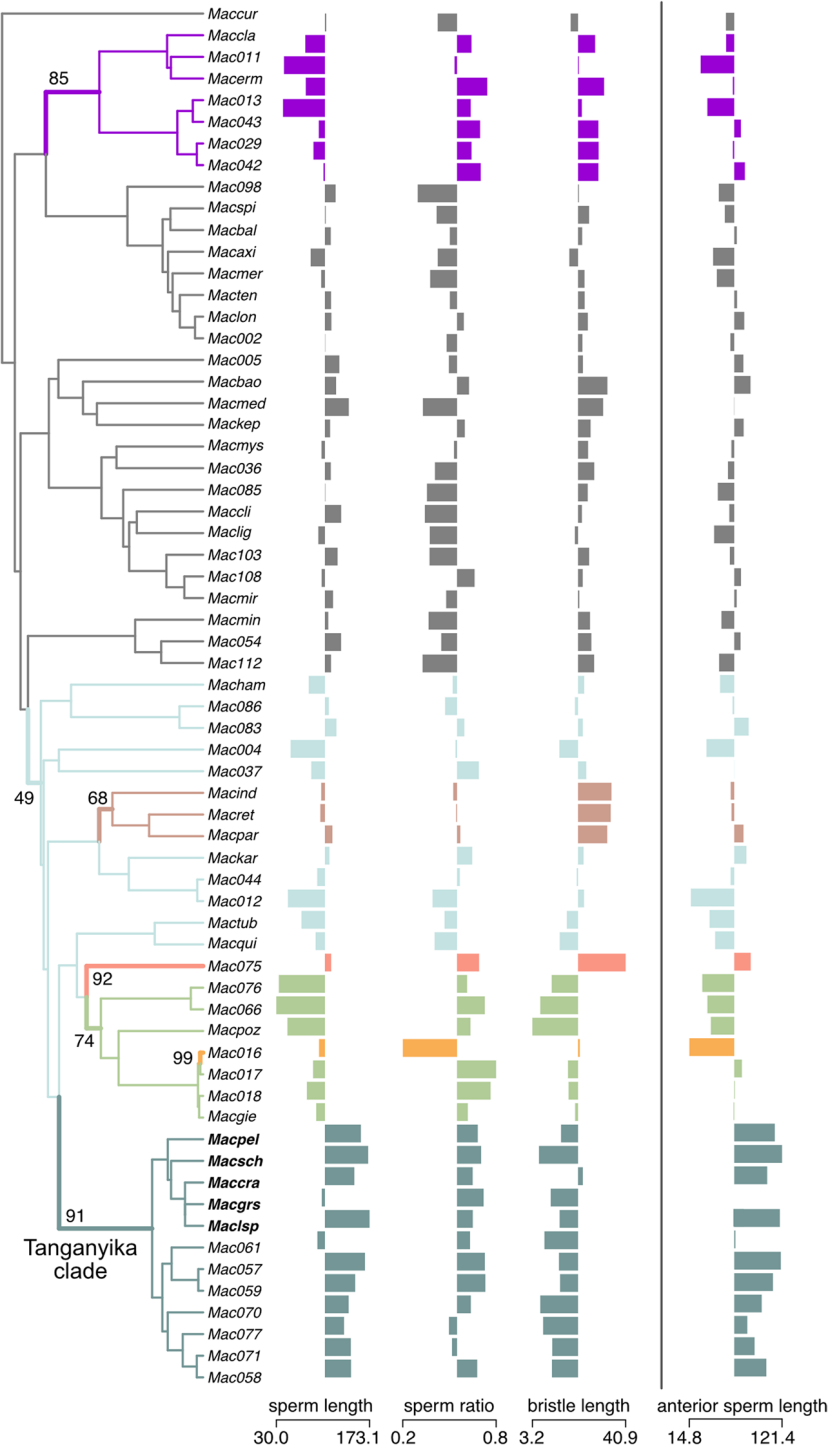
Phylogenetic analysis of sperm evolution using the R package *l1ou*. Included are 64 species assigned to the reciprocal mating syndrome. Sperm length, sperm ratio, and bristle length (all in µm)were included in the model, while anterior sperm length is only visualized but not included in the model. Shifts in Ornstein-Uhlenbeck optima are shown with color changes. Numbers indicate how often the shift was recovered out of 100 non-parametric bootstraps. Species names are abbreviated (See Additional file 1: Table S1), and the newly described species are highlighted in bold. Barplots show the standardized trait values. The numbers below indicate the unstandardized trait range. See Additional file 1: Table S1–S4, for the trait data and details on the inferred shifts

Most members of the Tanganyika clade had longer than average sperm compared to the average across the 64 species I measured (mean: 80.6 μm, SD: 31.8. range: 30.0–173.1 μm, Additional file 1: Table S2). Only the sperm of *M. gracilistylum* and *M.* sp. 61 were slightly shorter than average (Fig. 3). This difference was also statistically significant in the PGLS analysis (Additional file 1: Table S3). Similarly, the anterior sperm regions of the members of the Tanganyika clade were longer than average (mean: 46.0 μm, SD: 24.3, range:14.8–121.4 μm, Additional file 1: Table S2), which was again statistically significant in the PGLS analysis (Additional file 1: Table S3). It also appeared that the Tanganyika clade had an above-average sperm ratio, which in all five here described species exceeded 0.66 compared to the average 0.57 (mean: 0.57 μm, SD: 0.14, range: 0.22–0.82 μm, Additional file 1: Table S2). Furthermore, most species had shorter than average sperm bristles (mean: 12.8 μm, SD: 6.9, range: 3.2–40.9 μm, Additional file 1: Table S2). However, the PGLS analyses for the sperm ratio and sperm bristle length were not statistically significant (Additional file 1: Table S3).

These changes in sperm morphology were also reflected in the comparative analysis of sperm length, sperm ratio, and bristle length. The model with the lowest AICc value in the *l1ou* analysis indicated seven shifts in the evolutionary optima of sperm length, sperm ratio, and bristle length (Fig. 3). Bootstrap support for four of these seven shifts was low (below 90%), indicating low confidence, but the shifts on the branch to *M.* sp. 16, *M*. sp. 75, and the Tanganyika clade had high support (99%, 92%, and 91% respectively). Furthermore, the two alternative shift configurations (ΔAICc < 10 from the best model) also included the shift in *M.* sp. 16, *M.* sp. 75, and to the Tanganyika clade (Additional file 1: Table S4–S5, Additional file 2: Fig. S1). The detection of these shifts was, therefore, robust. The shift in *M.* sp. 16 was towards longer sperm, reduced sperm ratio, and increased bristle length. The shift in *M. sp.* 75 indicated an increase in all optima. Finally, the shift in the Tanganyika clade was towards longer sperm with an increased sperm ratio and shorter sperm bristles (Additional file 1: Table S4, Additional file 2: Fig. S1).

I also performed separate analyses only containing each trait individually. In the analysis including only total sperm length or only sperm ratios, every shift configuration contained a shift on the branch leading to the Tanganyika clade. Furthermore, this shift was the only one that was present in each configuration, indicating that it is robust (Additional file 1: Table S5, Additional file 2: Figs. S4–S5). In the analysis with only bristle length, all shift configurations contained a shift to shorter bristles on the branch leading to the tuba clade and Tanganyika clade, suggesting that the bristle reduction also occurrred in that sister clade (Additional file 1: Table S5, Additional file 2: Fig. S4). For sperm ratio analysis, the five top models had a mixed signal with two shift configurations indicating a shift in the Tanganyika clade and three shift configurations where a shift to a higher sperm ratio occurred deeper in the phylogeny (Additional file 1: Table S5, Additional file 2: Fig. S5). Finally, when I repeated the sperm length analysis while including species without sperm bristles (and thus no measurements for anterior sperm length, sperm ratio, and bristle length), I again found a shift on the branch leading to the Tanganyika clade in every analysis (Additional file 1: Table S5, Additional file 2: Fig. S6). In summary, the comparative analysis indicated shifts in the optimal trait values for the Tanganyika clade, showing a robust signal for increased total and anterior sperm length and a weaker signal for decreased bristle length and an increased sperm ratio.

### Species descriptions

To date, comparatively little taxonomic work on *Macrostomum* has been conducted on the African continent, especially compared to the extensive research done in Europe and North America [14, 41] and the Northern Hemisphere more generally [42]. Since recent results suggest that nearly all known freshwater species are descendants of a single evolutionary transition [13], I here primarily focus the taxonomic discussion to known African freshwater species. Moreover, given the widespread convergent evolution of morphological traits in the genus and the power of molecular phylogenetics, I largely focus our taxonomic discussion on species that have not yet been phylogenetically placed using molecular analyses [13] and also mention phylogenetically placed specimens when they are particularly similar.

With respect to African freshwater species, the most extensive work was conducted by Young [43], describing nine species from diverse freshwater habitats in Kenya, Tanzania, and Uganda. In addition, Young [43] also mentioned three species originally described from Europe. Moreover, Beltagi and colleagues [44–48] described eight species from freshwater, and in one case slightly brackish, habitats in the Nile Delta in Egypt. Furthermore, de Beauchamp [49] (often erroneously cited as 1935) reports on a species collected from freshwater on Mt. Elgon (Uganda) that was originally described as *M. viride* var. *elgonense* de Beauchamp, 1936. Ferguson [50] raised this variety to species level, naming it *Macrostomum beauchampi* Ferguson, 1939, but Ferguson [14] later corrected that, naming it *Macrostomum elgonense* (de Beauchamp, 1935), with which Marcus [51] concurs (but Young does not [52]). Since brackets are not needed when raising a subspecies to species, I refer to the species as *Macrostomum elgonense* de Beauchamp, 1936, to acknowledge the publication year.

Finally, Marcus and Marcus [53] described *M. tuba* var. *verbekei* Marcus & Marcus, 1957 from Goma, Lake Kivu, Democratic Republic of the Congo, which they named with reservation as a variety of *M. tuba*. Both Luther [41] and Young [43] noted that it seemed unlikely that this species is close to *M. tuba*. Given the consistently larger body and stylet size in *M. tuba* sampled across several continents [13], I concur with their assessment (and not with Reyes et al. [54], who, without further explanation, synonymize it with *M. tuba*) and therefore raise this variety to species level, designating it *Macrostomum verbekei* Marcus & Marcus, 1957. Note, however, that Young [43] reports on specimens of *M. tuba* that vary considerably in body size depending on the sampling site and location. Finally, Brand et al. [13] have reported several putative species from Egypt, Tunisia, and South Africa, but they have not been formally described.

Generally, it can be said that the five species described here do not share significant similarities with any of the previously described African species. In the following species descriptions, I will mention in the differential discussion when any of them might be similar.

### Family Macrostomidae Van Beneden, 1870

### Genus *Macrostomum* Schmidt, 1848

### *Macrostomum crassum*, sp. nov.

**Fig. 4:**
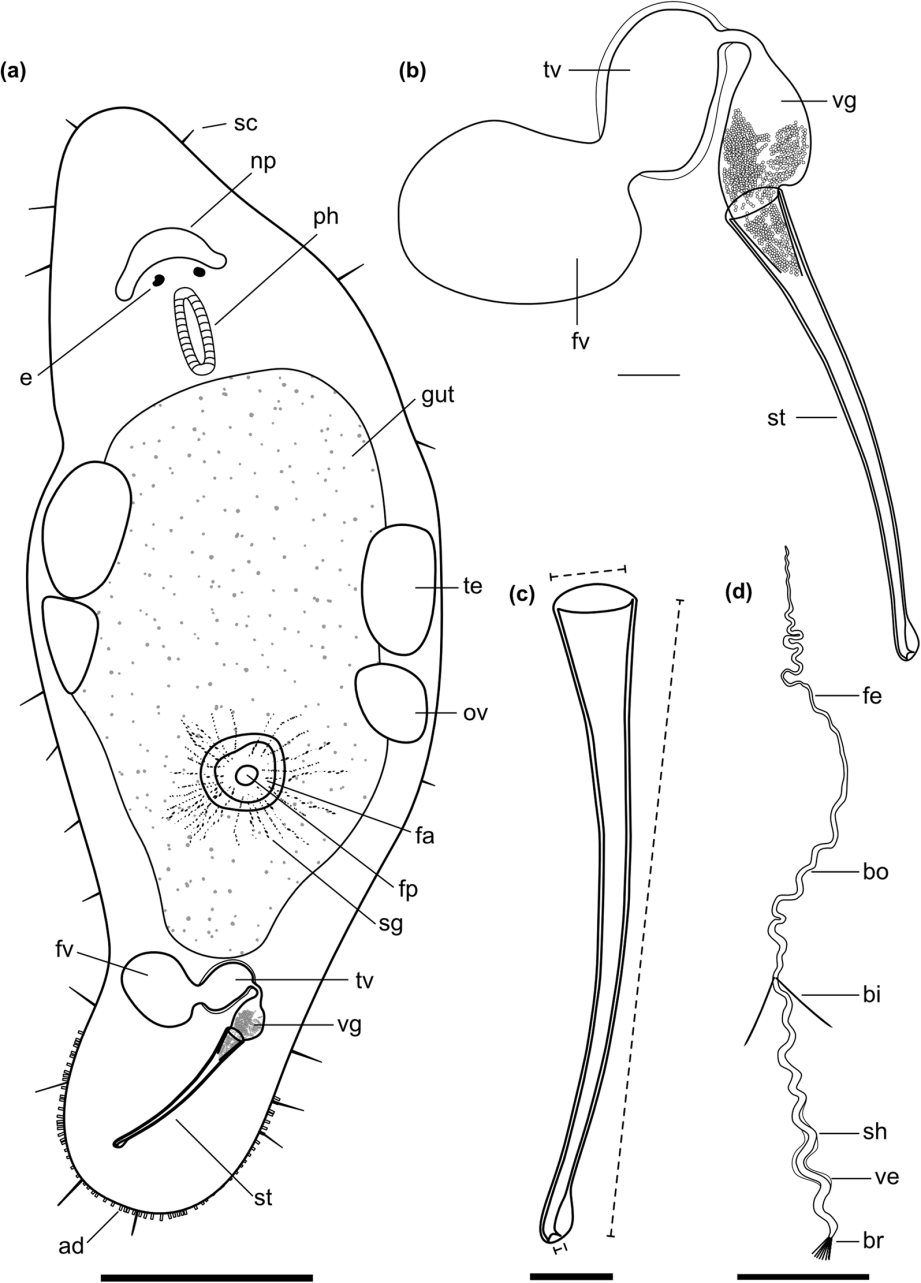
Line drawings of *Macrostomum crassum*. **a** Habitus (dorsal view and lightly squeezed), with sensory cilia (sc), neuropile (np), eyes (e), pharynx (ph), testis (te), ovary (ov), female gonopore (fp), female antrum (fa), shell glands (sg), and adhesive glands (ad). **b** Male genital system with false seminal vesicle (fv), muscular true seminal vesicle (tv), *vesicula granulorum* (vg), and stylet (st). **c** Detailed drawing of the stylet (indicated are the measurements for the straight stylet length and the width of the proximal and distal stylet openings, while the segmented stylet length (in Table 2) corresponds to the average length of both sides of the stylet). **d** Mature sperm cell with feeler (fe), body (bo), bristles (bi), shaft (sh), velum (ve), and brush (br). Note that it is difficult to distinguish the feeler and body region of the sperm in this species. Scale bars indicate 200 μm in (**a**) and 20 μm in (**b**–**d**)

**Fig. 5:**
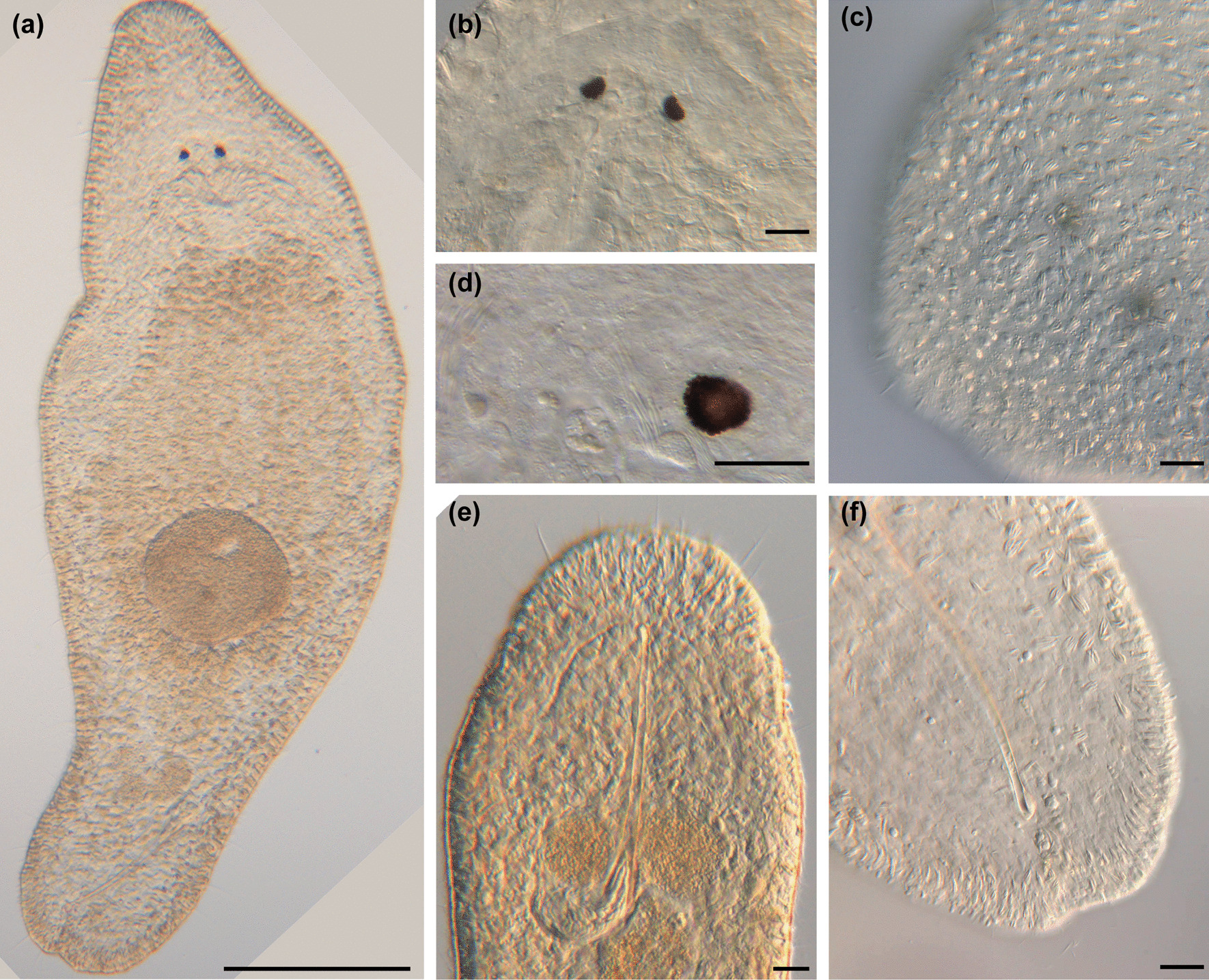
Micrographs of somatic structures of field-collected *Macrostomum crassum.* Brackets denote accessions of deposited specimens, with MTP LS 2644 being the HOLOTYPE. **a** Overview of an adult slightly squeezed worm (MTP LS 2521). **b** Eyes and neuropile (MTP LS 2644). **c** Dense rhabdites on the dorsal side of the rostrum (MTP LS 2644). **d** Detail of eye and rhammite glands (MTP LS 2521). **e** Caudal region with long sensory cilia (MTP LS 2644). **f** Tail plate with several rows of adhesive organs (MTP LS 2521). Scale bars indicate 200 μm in (**a**) and 20 μm in (**b**–**f**)

**Fig. 6:**
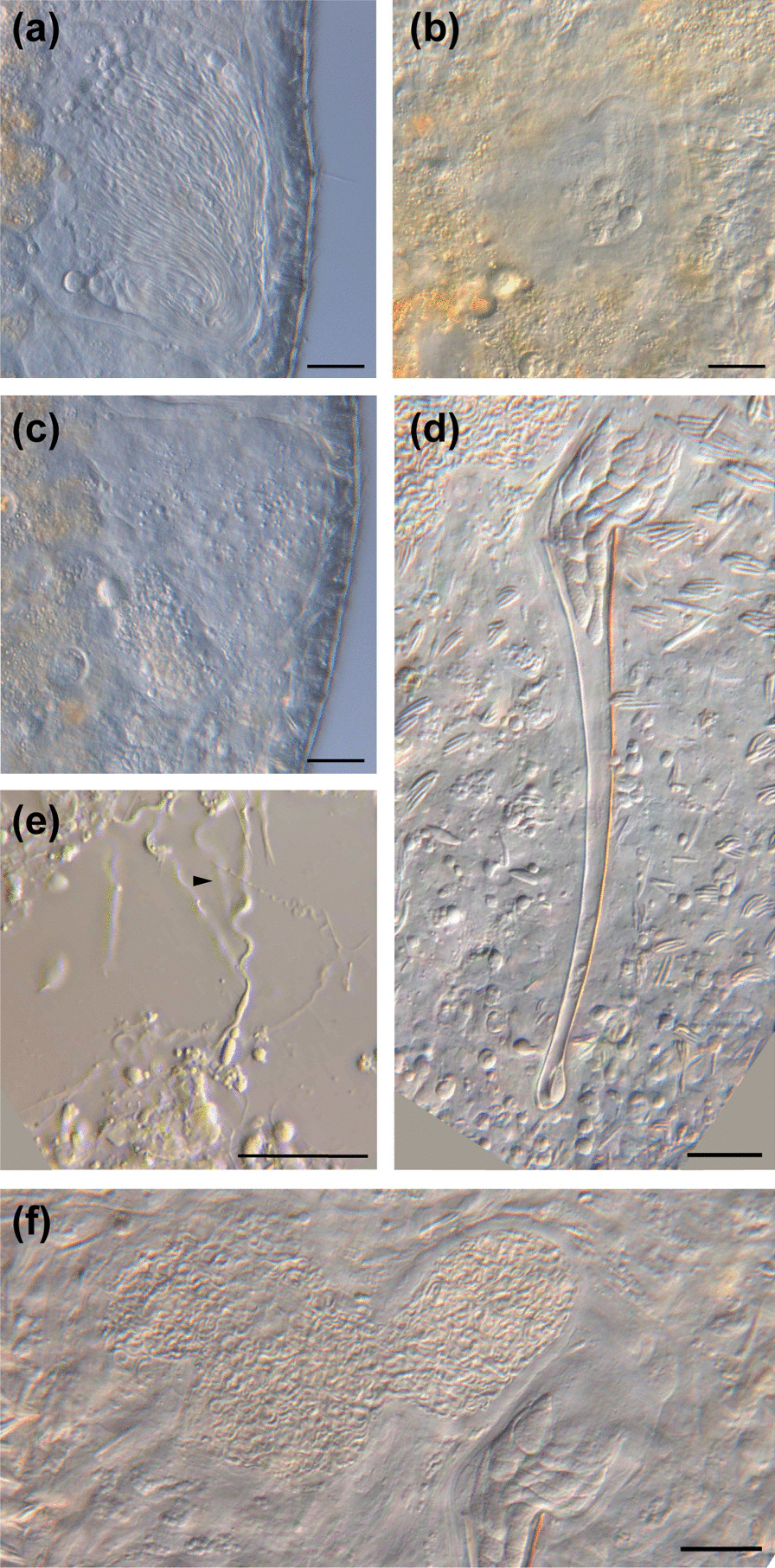
Micrographs of reproductive structures of *Macrostomum crassum*. Brackets denote accessions of deposited specimens, with MTP LS 2644 being the HOLOTYPE. **a** Ripe testis (MTP LS 2644). **b** Female antrum (MTP LS 2644). **c** Ripe ovary (MTP LS 2644). **d** Merged image of the stylet and *vesicula granulorum* (MTP LS 2521). **e** Several sperm among tissue debris, the arrow indicates the anterior region of a sperm (MTP LS 2644). **f** Overview of the male system (from left to right): false seminal vesicle, muscular true seminal vesicle, and *vesicula granulorum* (MTP LS 2521). Scale bars indicate 20 μm

urn:lsid:zoobank.org:act:49CF45F4-1B9B-42D4-804A-ED1E6E8EF173 (Figs. 4, 5 and 6; Table 2).

**Table 2:** Morphometric measurements of *Macrostomum crassum*, *M. pellitum*, and *M. longispermatum*

**Parameter**	*M. crassum*				*M. pellitum*				*M. longispermatum*			
	**Mean**	**SD**	**range**	**N**	**Mean**	**SD**	**range**	**N**	**Mean**	**SD**	**range**	**N**
Body L	921.9	125.3	833.3–1010.5	2	947.1	146.5	760.9–1231.0	14	1187.1	254.5	903.6–1608.9	9
Body W	362.9	21.5	347.7–378.1	2	225.4	43.6	171.1–320.5	14	328.0	128.5	172.1–550.6	8
Eye ø	12.5	2.5	10.7–14.3	2	11.1	1.1	9.1–12.5	7	14.3	1.1	13.3–15.9	6
Testis L	105.2	3.1	103–107.4	2	214.0	51.0	150.7–292.8	12	202.5	30.4	154.3–237.4	5
Ovary L	86.7	1.1	86–87.5	2	63.1	21.5	32.6–105.4	12	110.3	54.6	66.7–179.7	5
Short sensory cilia L	12.8	7.8	7.3–18.3	2	10.7	3.2	8.3–19.7	12	10.9	3.4	7.1–16.9	6
Long sensory cilia L	20.8	4.2	17.9–23.8	2	28.5	3.5	23.8–35.7	13	18.8	4.3	13.4–26.0	7
Rhabdite L	9.3	1.2	8.5–10.1	2	11.0	2.9	8.8–16.7	7	9.4	2.0	7.2–11.1	3
Stylet traits												
Straight L	152.0	2.0	150.6–153.5	2	86.1	5.1	80.7–96.7	9	138.4	4.1	134.3–146.1	7
Segmented L	156.1	1.9	154.8–157.4	2	79.5	4.8	73.6–88.9	9	152.4	19.3	107.9–172.6	8
Proximal opening W	17.4	0.9	16.7–18.0	2	12.0	1.3	9.6–13.6	9	11.5	1.4	9.5–13.7	8
Distal opening W	4.0	0.3	3.8–4.2	2	6.9	1.1	4.4–8.1	9	2.7	0.2	2.4–3.1	8
Sperm traits												
Total L	130.0	–	–	1	147.1	0	147.1–147.1	2	173.1	9.2	166.6–179.6	2
Anterior L	86.7	–	–	1	102.9	0.4	102.6–103.1	2	115.5	1.4	114.6–116.5	2
Bristle L	12.7	–	–	1	7.0	3.0	4.0–10.1	3	6.8	1.1	5.5–7.4	3
Brush L	3.8	–	–	1	4.2	0	4.2–4.2	2	3.7	0.3	3.5–3.9	2

All length, width, and diameter measurements are in µm. L: length, W: width, ø: diameter

#### Material examined

Holotype: One digitally-documented specimen (MTP LS 2644), including 54 images and videos and a whole mount (Table 1), from the type locality collected on 26 August 2016 from aquatic plants (*Vallisneria* sp.) with roots at 1 m depth and 2 m from the shore in Lake Tanganyika, Zambia. Paratype: One digitally-documented specimen (MTP LS 2521), including 31 images and videos, a partial *28 S rRNA* sequence, and a whole-body transcriptome from an earlier sample collected at the type locality on 20 August 2016.

#### Etymology

The name derives from the Latin *crassum*, meaning stout, which refers to this species’ compressed appearance along the anterior-posterior axis. *M. crassum* has previously been referred to as *Macrostomum* sp. 60 or Mac060.

#### Diagnosis

*Macrostomum* with a conical rostrum; body length between 833.3 and 1010.5 μm (see also Table 2); small kidney-shaped eyes; long sensory cilia along body; testes clearly bigger than ovaries; ovaries immediately posterior of testes; false seminal vesicle without musculature; true seminal vesicle with muscular wall; true and false vesicle at similar height; *vesicula granulorum* large with prominent gland necks reaching into stylet base; stylet with narrow funnel-shaped base and long sweeping shaft; asymmetrical distal stylet thickening with a rounded drop on the shorter side and an oblique opening; sperm with an unusually long anterior sperm length, two bristles, slight vellum, and brush.

Description (see also Table 2).

#### General morphology

A tongue-shaped *Macrostomum* with a conical rostrum and a broad tail plate. The sensory cilia are long and visible all around the body margin (Figs. 4a and 5a and e). The eyes are kidney-shaped and lie just anterior to the pharynx (Fig. 5b, d). The body is densely covered in bundles of 3–7 rhabdites, which are especially visible along the body margin (Fig. 5a, c, f). The gut extends up to 78% body length (BL), terminating at the seminal vesicles.

#### Male system

The testes are medium to large, starting at 29% BL and extending to 50% BL, and have clear bundles of elongating sperm at the posterior end leading to the vas deferens (Fig. 6a). The *vasa deferentia* join in a false seminal vesicle without visible musculature. The true seminal vesicle has a muscular wall and is similar in size to the false vesicle. The false and true seminal vesicles are on the same level at 80% BL and, depending on the stylet position can be anterior or posterior to the stylet base (Fig. 6f). The true seminal vesicle connects via the ductus intervesicularis to a large *vesicula granulorum* that reaches into the stylet base and contains prominent prostate secretion granules (Figs. 4b and 6d). The stylet is a long tube with a narrowing funnel-shaped base, which then remains similarly narrow for much of the rest of the stylet. The stylet has a distal asymmetrical thickening with a large, rounded drop on the shorter side and a slightly oblique opening (Fig. 4c). The male pore is far posterior at 95% BL. The sperm is long with an unusually long anterior sperm length, two bristles, a shaft with a slight vellum or ridge, and a brush (Fig. 4d).

#### Female system

The ovaries are immediately posterior to the testes at 50% BL and extend to 56% BL (Fig. 6c). The vagina is central at 60% BL and has distinct radially symmetrical shell glands. The epithelium of the vagina is heavily ciliated and thickened, leading into a ciliated antrum chamber with a thick epithelium and a thick cellular valve (Fig. 6b). No received sperm was observed in the two collected specimens.

#### Discussion

The large asymmetrical thickening of the distal stylet opening on a fairly straight stylet is unique among the described *Macrostomum* species. *M. crassum*’s stylet is similar to two of the undescribed members of the Tanganyika clade, namely *M*. sp. 61 and *M*. sp. 69, however, the thickening is much more symmetrical in those species, and they are clearly molecularly diverged [13].

### *Macrostomum pellitum*, sp. nov.

**Fig. 7:**
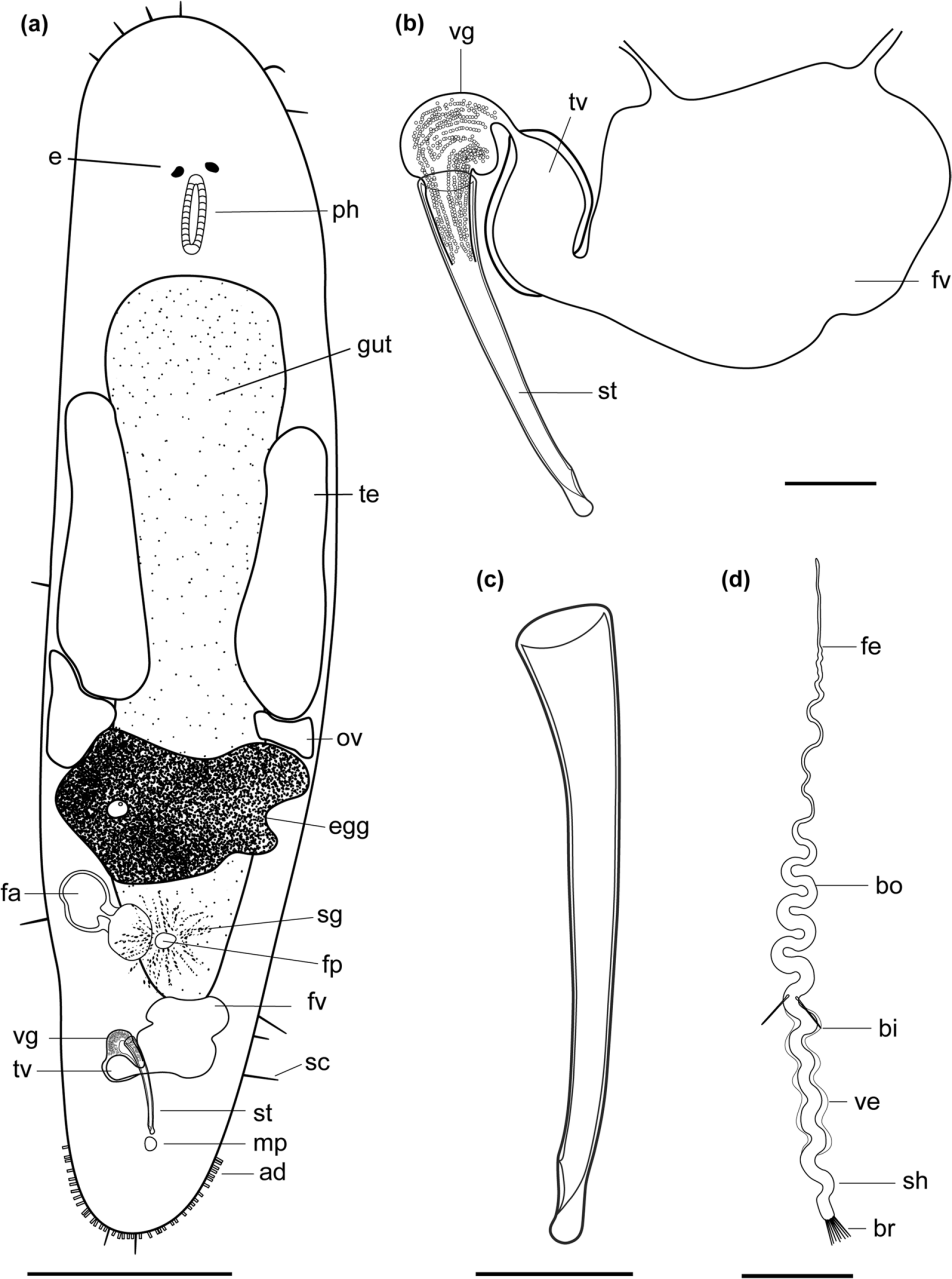
Line drawings of *Macrostomum pellitum*. **a** Habitus (dorsal view and lightly squeezed), with sensory cilia (sc), eyes (e), pharynx (ph), testis (te), ovary (ov), female gonopore (fp), female antrum (fa), shell glands (sg), male gonopore (mp), and adhesive glands (ad). **b** Male genital system with false seminal vesicle (fv), muscular true seminal vesicle (tv), *vesicula granulorum* (vg), and stylet (st). **c** Detailed drawing of the stylet (indicated are the measurements for the straight stylet length and the width of the proximal and distal stylet openings, while the segmented stylet length (in Table 2) corresponds to the average length of both sides of the stylet. **d** Mature sperm cell with feeler (fe), body (bo), bristles (bi), velum (ve), shaft (sh), and brush (br). Note that it is difficult to distinguish the feeler and body region of the sperm in this species. Scale bars indicate 100 μm in (**a**) and 20 μm in (**b**–**d**)

**Fig. 8:**
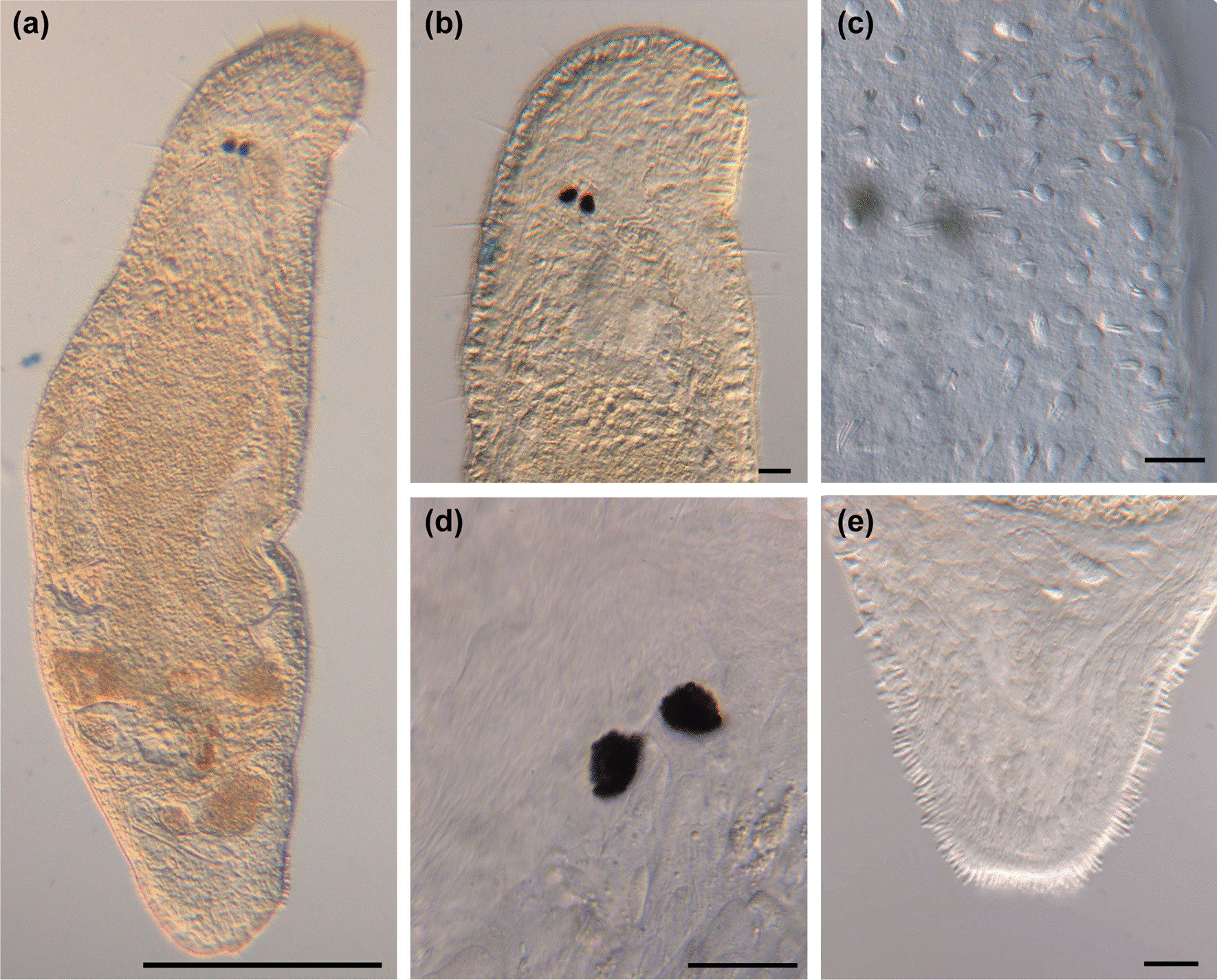
Micrographs of somatic structures of field-collected *Macrostomum pellitum*. Brackets denote accessions of deposited specimens, with MTP LS 2600 being the HOLOTYPE. **a** Overview of an adult slightly squeezed worm (MTP LS 2600). **b** Close-up of the anterior region with abundant long cilia (MTP LS 2600). **c** Rhabdites (MTP LS 2737). **d** The characteristically close-set pigment cup eyes (MTP LS 2585). **e** The small tail plate with adhesive organs (MTP LS 2600). Scale bars indicate 100 μm in (**a**) and 20 μm in (**b**–**e**)

**Fig. 9:**
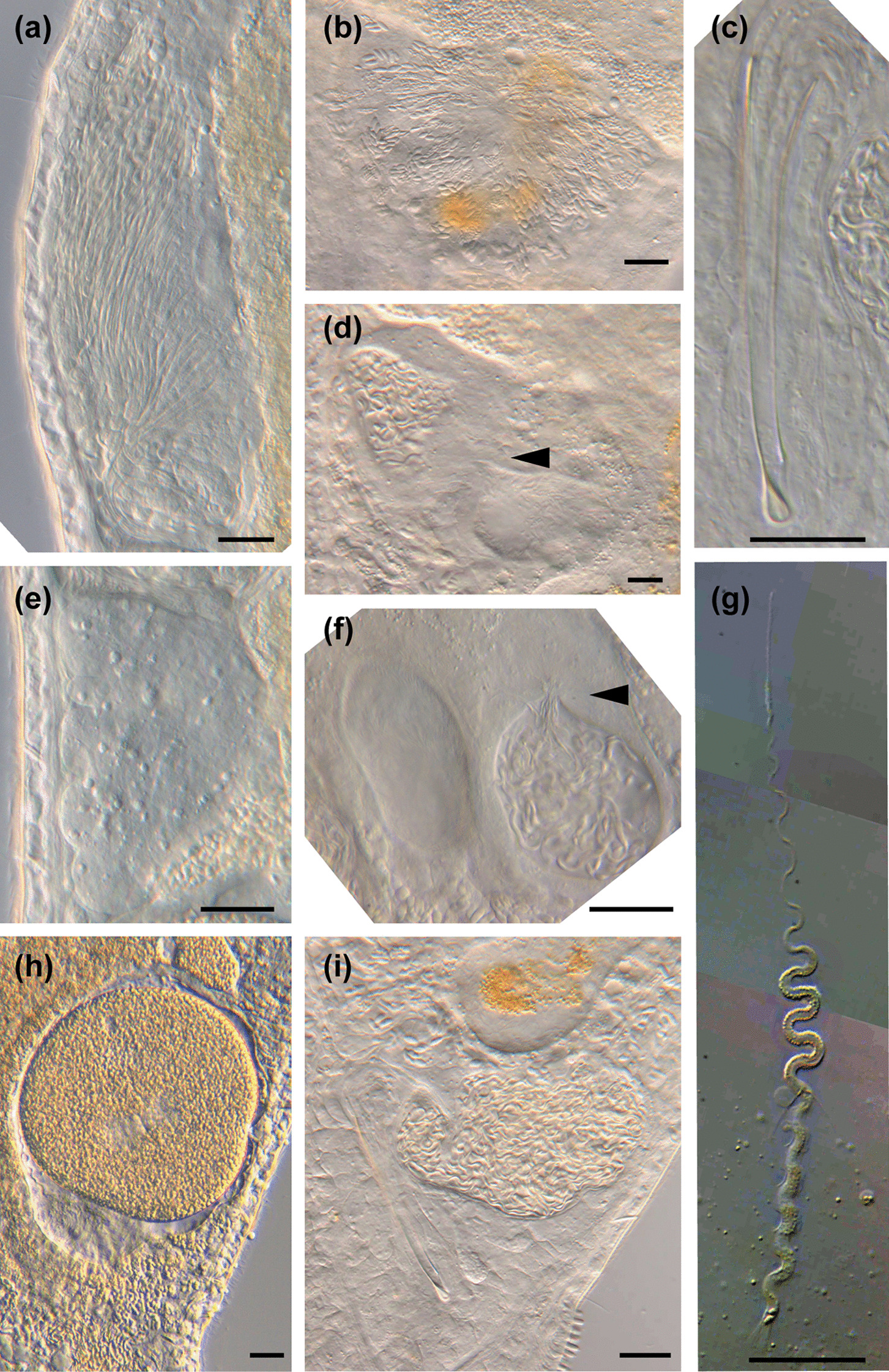
Micrographs of reproductive structures of field-collected *Macrostomum pellitum*. Brackets denote accessions of deposited specimens, with MTP LS 2600 being the HOLOTYPE. **a** Ripe testis (MTP LS 2737). **b** Shell glands surrounding the vagina (MTP LS 2600). **c** The stylet with the parts of the *vesicula granulorum* extending into its base (MTP LS 2600). **d** Female antrum with the ciliated first chamber in the lower right and the thickened second chamber in the upper left. The second chamber is filled with sperm, and the connection between the chambers is indicated (arrow, MTP LS 2600). **e** Ripe ovary (MTP LS 2737). **f** Female antrum, showing an empty first chamber (left) and the second chamber (right) filled with sperm, sperm feelers of several sperm are embedded in the antrum wall (arrow, MTP LS 2617). **g** Composite image of a sperm, showing the short sperm bristles and the terminal brush (MTP LS 2740). **h** An egg during the first mitosis (note the two nuclei, arrow) in the antrum, surrounded by sperm (open arrow, MTP LS 2733). **i** Overview of the male system with stylet, *vesicula granulorum*, and both seminal vesicles (MTP LS 2600). Scale bars indicate 20 μm

urn:lsid:zoobank.org:act:A7E66372-F20D-4E05-9F09-684552277194 (Figs. 7, 8 and 9; Table 2).

#### Material examined

Holotype: One digitally-documented specimen (MTP LS 2600), including 45 images and videos and a whole mount (Table 1), from the type locality collected on 24 August 2016 from coarse red sand of approximately 1–2 mm grain size at the waterline on the beach close to Mjamba village, Lake Tanganyika, Zambia. Paratypes: 15 digitally-documented specimens, including a total of 384 images and videos, two partial *28 S rRNA* sequences, one whole-body transcriptome, and three whole-mounts (Table 1), comprised of eight specimens from the original sample and seven specimens from a later sample of the type locality collected on 3 September 2016. Other material: three digitally-documented specimens, including 93 images and videos, from a nearby locality collected on 23 August 2016 from the sand below the waterline on the beach in Isanga Bay, Lake Tanganyika, Zambia.

#### Etymology

From the Latin *pellitus*, meaning covered with skins or furs, referring to the ubiquitous long sensory cilia. *M. pellitum* has previously been referred to as *Macrostomum* sp. 65 or Mac065.

#### Diagnosis

*Macrostomum* with tongue-shaped body; body length between 760.9 and 1231.0 μm (Table 2); long sensory cilia along the entire body; two small pigment cup eyes close together; gut extends caudally beyond the female antrum; testes central and considerably larger than ovaries; vagina central, leading to a two-chambered antrum; first antrum chamber thin-walled and covered in cilia, connected to a second muscular chamber via a muscular duct; stylet an almost straight, gradually narrowing funnel terminating in a wide subterminal opening orthogonal to the stylet axis; stylet tip with a single large drop-shaped distal thickening; sperm with no distinct feeler, but short bristles, thick brush and thin velum posterior of the bristles.

Description (see also Table 2).

#### General morphology

A tongue-shaped *Macrostomum* lacking a distinct tail plate (Figs. 7a and 8a). Long sensory cilia are abundant along the entire body (Fig. 8a–b), and stouter and shorter cilia are present along the rostrum. The two small kidney-shaped pigment eyes lie close together (Fig. 8d) just anterior of the unremarkable pharynx. The gut extends caudally beyond the female antrum, terminating at the false seminal vesicle at 83% BL.

#### Male system

The large testes begin at 36% BL and extend to 60% BL. Bundles of sperm are clearly visible at the posterior end of the testes curving towards the *vasa deferentia* (Fig. 9a). The *vasa deferentia* join in a large false seminal vesicle without visible musculature (Figs. 7b and 9i). The generally large false seminal vesicle leads to a small muscular true seminal vesicle, which lies lateral or sometimes posterior to the stylet base. The true seminal vesicle connects to a *vesicula granulorum*, with the gland necks of the *vesicula granulorum* extending far into the stylet (Figs. 7b and 9c). The stylet is an almost straight, gradually narrowing funnel terminating in a wide subterminal opening orthogonal to the stylet axis. Distally the stylet is terminated by a single large drop-shaped thickening (Figs. 7c and 9c). The unremarkable male pore is at 92% BL. The stylet can change position and is then positioned approximately between the true and false seminal vesicle. The sperm is long with a long thin region anterior to the short bristles but with no clear transition between the sperm body and the sperm feeler. Posterior to the bristles, sperm has a thin velum and terminates in a thick brush (Figs. 7d and 9g).

#### Female system

The paired, small ovaries lie immediately posterior to the testes, sometimes overlapping with them on the side (Figs. 7a and 8a). The ovaries start at 59% BL and extend to 64% BL. Developing eggs are frequently observed centrally, slightly posterior to the ovaries. The vagina lies centrally at 77% BL and is surrounded by sparse, radially arranged shell glands (Fig. 9b). The vagina leads to a two-chambered antrum, which in slightly squeezed specimens is displaced from the midline either to the right (e.g., MTP LS 2587) or to the left (e.g., MTP LS 2600, Fig. 8a). The first antrum chamber is completely covered in cilia with a thin wall initially but with strong musculature at the wall towards the second chamber. The chambers are connected via a muscular duct that can be closed completely (Fig. 9d). The second chamber has a thick epithelium, where sperm is often seen embedded with their feelers in the wall, oriented towards the developing eggs (Fig. 9f). Most specimens (17/19) had sperm in the second chamber, but only two also had sperm in the first chamber. When an egg enters the antrum, the muscular duct between the chambers appears to open to accommodate it (Fig. 9h).

#### Discussion

Among the African freshwater species *M. pellitum* has some superficial similarity with *Macrostomum christinae* Young, 1976, since both have a straight stylet. However, the stylet of *M. christinae* has a terminal opening, no strong distal thickening, and is shorter (72 μm). Among other *Macrostomum* species, *M. pellitum* is similar to *M. obtusa* because it has a very similar stylets. Both stylets have a subterminal opening, a pronounced anterior thickening, and a similar length (82–91 μm for *M. obtusa* versus 81–97 μm). However, both species are clearly distinct in the *28 S rRNA* sequence [13], suggesting the similarity is due to convergent evolution. Additionally, several characteristics distinguish the species. First, the seminal vesicles and the *vesicula granulorum* are arranged in a line in *M. obtusa*, while they are next to each other in *M. pellitum*, as is more typical for the genus. Second, the antrum of *M. obtusa* is simple and lies centrally, while it has two chambers in *M. pellitum*, one of which shows a clear muscular wall that is usually laterally displaced. Naturally, because of its similarity with *M. obtusa*, *M. pellitum* also has some resemblance to *M. tenuicauda*, but it is clearly distinct because the stylet of *M. tenuicauda* is shorter (56–75 μm).

### *Macrostomum longispermatum*, sp. nov.

**Fig. 10:**
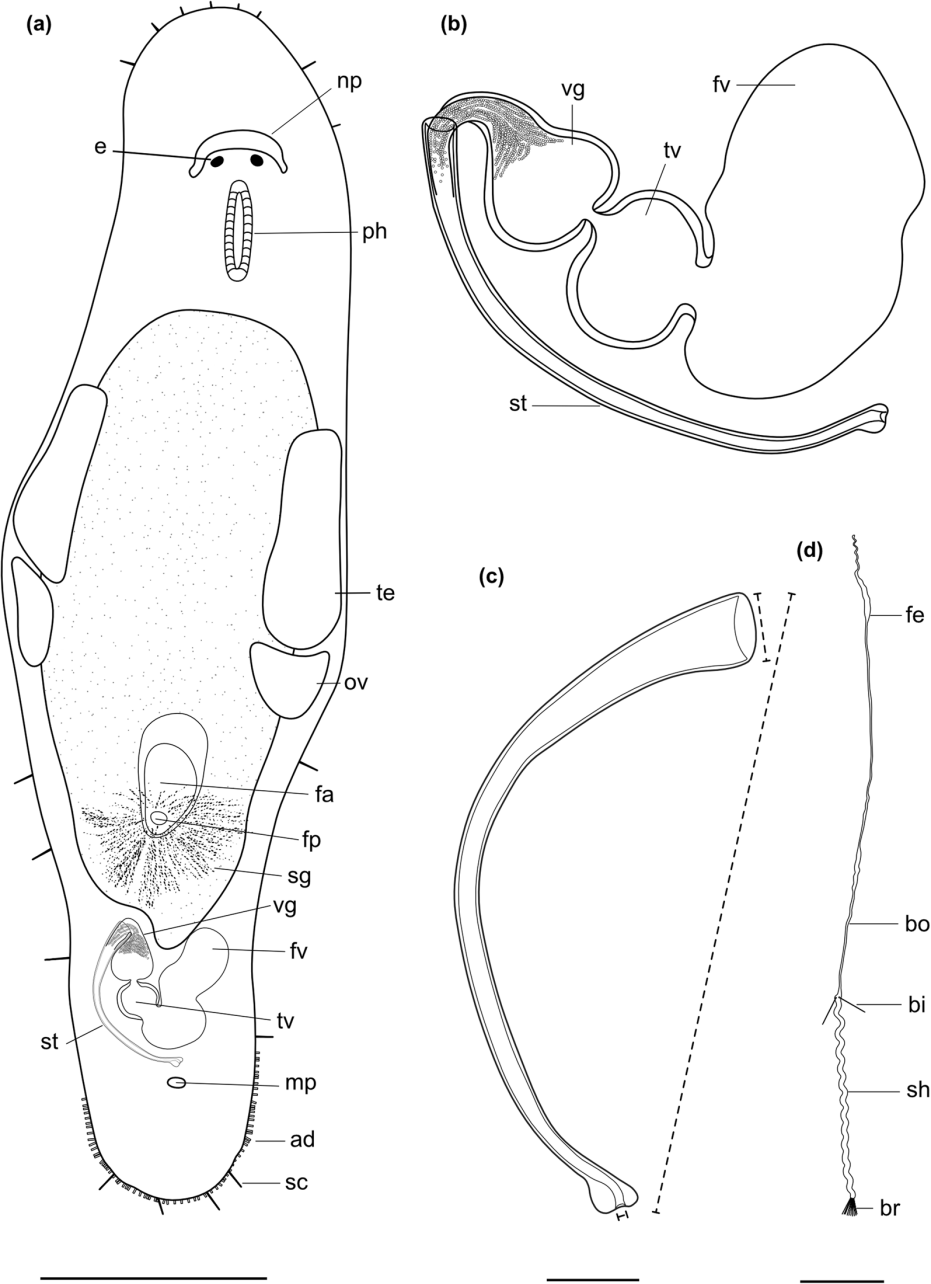
Line drawings of *Macrostomum longispermatum*. **a** Habitus (lightly squeezed), with sensory cilia (sc), eyes (e), pharynx (ph), testis (te), ovary (ov), female gonopore (fp), female antrum (fa), shell glands (sg), male gonopore (mp), and adhesive glands (ad). **b** Male genital system with false seminal vesicle (fv), muscular true seminal vesicle (tv), *vesicula granulorum* (vg), and stylet (st). **c** Detailed drawing of the stylet (indicated are the measurements for the straight stylet length and the width of the proximal and distal stylet openings, while the segmented stylet length (in Table 2) corresponds to the average length of both sides of the stylet). **d** Mature sperm cell with feeler (fe), body (bo), bristles (bi), shaft (sh), and brush (br). Note that it is difficult to distinguish the feeler and body region of the sperm in this species. Scale bars indicate 100 μm in (**a**) and 20 μm in (**b**–**d**)

**Fig. 11:**
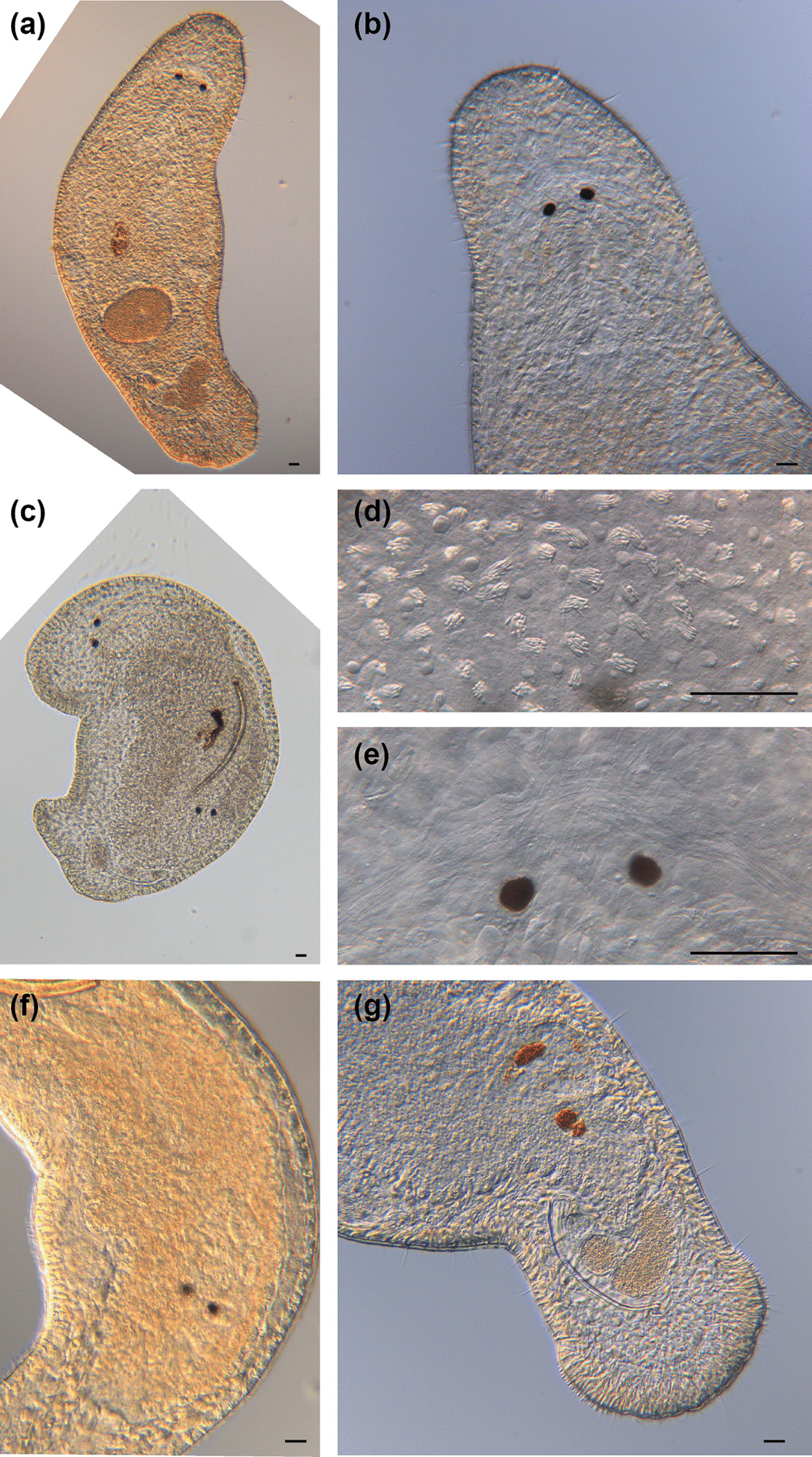
Micrographs of somatic structures of *Macrostomum longispermatum*. Brackets denote accessions of deposited specimens, with MTP LS 2598 being the HOLOTYPE. **a** Overview of an adult slightly squeezed worm (MTP LS 2698). **b** Anterior region with short sensory cilia (MTP LS 2598). **c** This specimen has ingested another flatworm, potentially also a *Macrostomum* species (MTP LS 2684). **d** Abundant rhabdites on the ventral epidermis (MTP LS 2683). **e** Eyes and neuropile (MTP LS 2683). **f** Detail of (c). **g** Caudal region showing the distinct tail plate with multiple rows of adhesive organs (MTP LS 2613). Scale bars indicate 20 μm

**Fig. 12:**
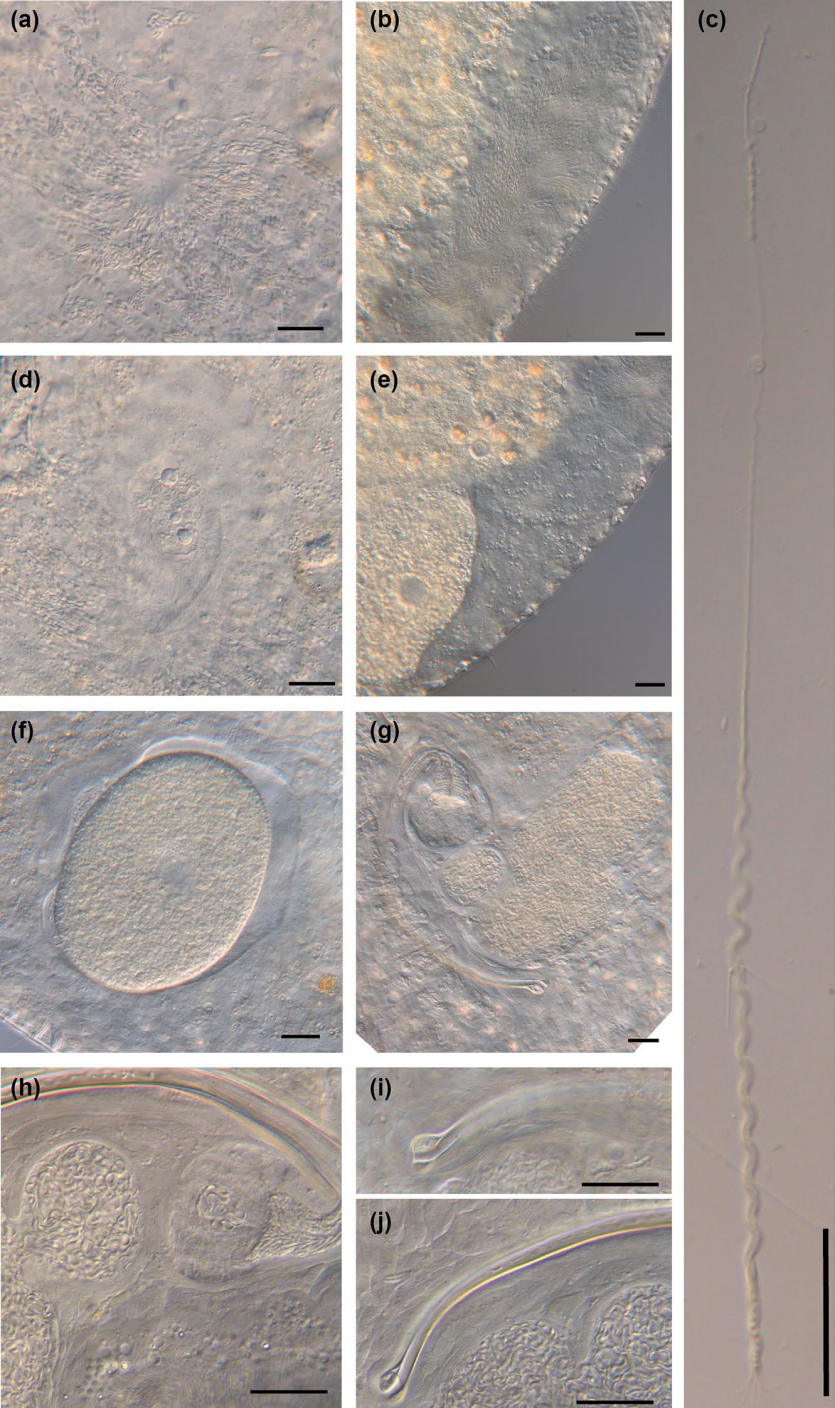
Micrographs of reproductive structures of field-collected *Macrostomum longispermatum*. Brackets denote accessions of deposited specimens, with MTP LS 2598 being the HOLOTYPE. **a** Vagina with shell glands (MTP LS 2695). **b** Ripe testis with bundles of elongating sperm (MTP LS 2683). **c** Stitched image of a sperm with bristles and a brush (MTP LS 2613). **d** Female antrum containing sperm, note the thick anterior epithelium (MTP LS 2695). **e** Ripe ovary and parts of a developing egg (MTP LS 2683). **f** Egg and sperm inside the female antrum (MTP LS 2598). **g** Overview of the male system with stylet, *vesicula granulorum*, and both seminal vesicles (MTP LS 2613). **h** Detail of the true seminal vesicle and the *vesicula granulorum*. Note the sperm inside the *vesicula granulorum* (MTP LS 2613). **i** Detail of the distal stylet thickening (MTP LS 2598). **j** Another detail of the distal stylet thickening. Note the difference in the shape of the larger part of the thickening (MTP LS 2613). Scale bars indicates 20 μm

urn:lsid:zoobank.org:act:EBA62FCC-B34B-47F6-BBEB-ED8F802F932A (Figs. 10, 11 and 12; Table 2).

#### Material examined

Holotype: A digitally-documented specimen (MTP LS 2598), including 61 images and videos and a whole mount (Table 1), from the type locality collected on 24 August 2016 from coarse red sand of approximately 1–2 mm grain size at the waterline on the beach close to Mjamba village, Lake Tanganyika, Zambia. Paratypes: nine digitally-documented specimens, including 338 images and videos, a *28 S rRNA* fragment, a whole-body transcriptome, and three whole mounts from the original sample at the type locality. Other material: one digitally-documented specimen (MTP LS 2626), including 46 images and videos, and a whole mount (Table 1), collected from aquatic plants and grasses at the mouth of the Kalambo River, Lake Tanganyika, Zambia. One digitally-documented specimen (MTP LS 2695), including 28 images and videos, was collected from the base of reeds in the surf zone of a rocky shore, Lake Tanganyika, Zambia.

#### Etymology

The name refers to this species’ unusually long sperm. *M. longispermatum* has previously been referred to as *Macrostomum* sp. 67 or Mac067.

#### Diagnosis

*Macrostomum* without a very distinct tail plate; body length between 903.6 and 1608.9 μm (see also Table 2); oval eyes; testes larger than ovaries; elongating sperm visible in testes lumen with a distinct curve towards the *vasa deferentia*; false seminal vesicle without musculature located anterior of the muscular true seminal vesicle; onion-shaped *vesicula granulorum* muscular with large ciliated proximal lumen and dense distal rows of granules; *vesicula granulorum* extending somewhat into stylet base; stylet with a long sweeping curve ending with a 45º curve to the convex side; stylet base conical, narrowing after ~ 30% of the length and narrowing further at ~ 90%; rounded distal thickenings, about twice as wide on the convex side; sperm with brush and short bristles; unusually long anterior of variable width; ovaries immediately posterior to testes; eggs often quite small; shell glands extending in a fan pattern posterior and only slightly anterior; antrum ciliated with a clear wall and anterior cellular valve.

Description (see also Table 2).

#### General morphology

A relatively slender *Macrostomum* without a distinct tail plate and oval pigment cup eyes (Figs. 10a and 11a). Sensory cilia are found all over the body but are most pronounced on the rostrum and posterior to the ovaries (Fig. 11b, g). The gut extends posteriorly to the margin of the false-seminal vesicle at 81% BL. Thick rows of adhesive organs are arranged on the tail plate (Fig. 11g).

#### Male system

The large testes start at 35% BL and extend to 53% BL. Bundles of elongating sperm are clearly visible in the testis lumen at the posterior end of the testes curving towards the *vasa deferentia* (Fig. 12b). *Vasa deferentia* joins in a large false seminal vesicle without apparent musculature. While the false seminal vesicle consists of one chamber, in some specimens, it has a prominent constriction in the middle, giving it a dumbbell shape (Fig. 12g). The small, muscular true seminal vesicle extends anteriorly from the false seminal vesicle through a wide ductus intervesicularis (Figs. 10b and 12h). The muscular *vesicula granulorum* extends again anterior from the true seminal vesicle and has a large ciliated proximal lumen similar in size to the true seminal vesicle, which often contains some sperm (Fig. 12g & h). The distal part of the *vesicula granulorum* has dense rows of granules extending into the base of the stylet (Fig. 10b). The stylet describes a long sweeping curve almost completing a semi-circle and then a 45º curve to the convex side. The stylet base is conical but then narrows to an intermediate diameter after ~ 30% of its length, then maintains this diameter until it finally narrows again towards the distal opening at ~ 90% of its length (Figs. 10c and 12g). The stylet lies parallel to the line along the seminal vesicles and *vesicula granulorum*, but depending on the squeeze preparation, it can either be displaced laterally (e.g., MTP LS 2598, as drawn in Fig. 10a) or on top of the vesicles (e.g., MTP LS 2612). The distal stylet opening is surrounded by a rounded distal thickening roughly twice as wide on the convex side (Figs. 10c and 12i and j). The sperm has an unusually long anterior, which in some specimens has a region of increased thickness at ~ 20 μm. There are two short bristles at ~ 70% length and a terminal brush (Figs. 10d and 12c).

#### Female system

The small ovaries lie immediately posterior to the much larger testes (Figs. 10a and 12e). Developing eggs migrate posteriorly towards the central antrum. The eggs are quite small. The vagina lies at 68% BL. Shell glands are numerous and small, extending in a fan-like pattern posterior and only slightly anterior of the female opening (Fig. 12a). The antrum is ciliated with a clear wall that thickens anteriorly into an unusually large cellular valve (Fig. 12d). Sperm was observed in the antrum of half the studied specimen (6/12) between the cellular valve and an egg (5 cases) or in the anterior half (1 case). The sperm feeler did not appear to be embedded in the cellular valve.

#### Discussion

Among the African freshwater species, *M. longispermatum*’s stylet is similar to the stylet of *Macrostomum ismailiensis* Beltagi, Ibrahim & Moustafa, 2001, but that species’ stylet is longer (180 μm), and the distal thickening is not as pronounced. Since there are no sperm images available for *M. ismailiensis*, it is not possible to assess if it also possesses the characteristic long sperm. The stylet of *M. longispermatum* also has some similarity with a few stylets of undescribed members of the Tanganyika clade, especially *M.* sp. 58 and *M.* sp. 57 [13], but in both of these, the curve of the stylet is less pronounced, and the distal thickening is not as strong.

Among all other *Macrostomum*, *M. longispermatum* has some similarities with *M. tuba* Graff, 1882 and *M. subterraneum* Rixen, 1961. *M. tuba* also has a curved stylet with a distal thickening. However, the stylet of *M. longispermatum* is shorter, *M. tuba* is a much larger animal, and the sperm of *M. tuba* is much shorter (48 μm vs. 173 μm). The stylet of *M. longispermatum* has some similarity to *M. subterraneum* since both describe a large curve and have an asymmetrical thickening. However, *M. subterraneum*’s stylet is shorter (92 μm vs. 139 μm). Additionally, *M. subterraneum* has reduced eyes with little pigment, while they are well-developed in *M. longispermatum*. Finally, *M. subterraneum* appears to have a laterally displaced, two-chambered female antrum (As can be seen in Fig. 25 in [55], although it is not discussed in the text), while the antrum in *M. longispermatum* is central and quite simple.

#### Remarks

*M. longispermatum* appears to be a predator of other flatworms, perhaps even preying on *Macrostomum*, as evidenced by our observations of a small worm completely inside specimen MTP LS 2684 (Fig. 11c & f). While sperm feelers were not observed embedded in the cellular valve, the correlation of a thick cellular valve and unusually long anterior sperm region (see discussion) suggests they do embed. The lack of observation could be due to the presence of an egg in the antrum in most cases. If it had entered the antrum recently, it could have dislodged any embedded sperm. Discounting the specimens with an egg in the antrum leaves only a single observation. More detailed observations of this species are needed to determine how the sperm and cellular valve interact.

### *Macrostomum schareri*, sp. nov.

**Fig. 13:**
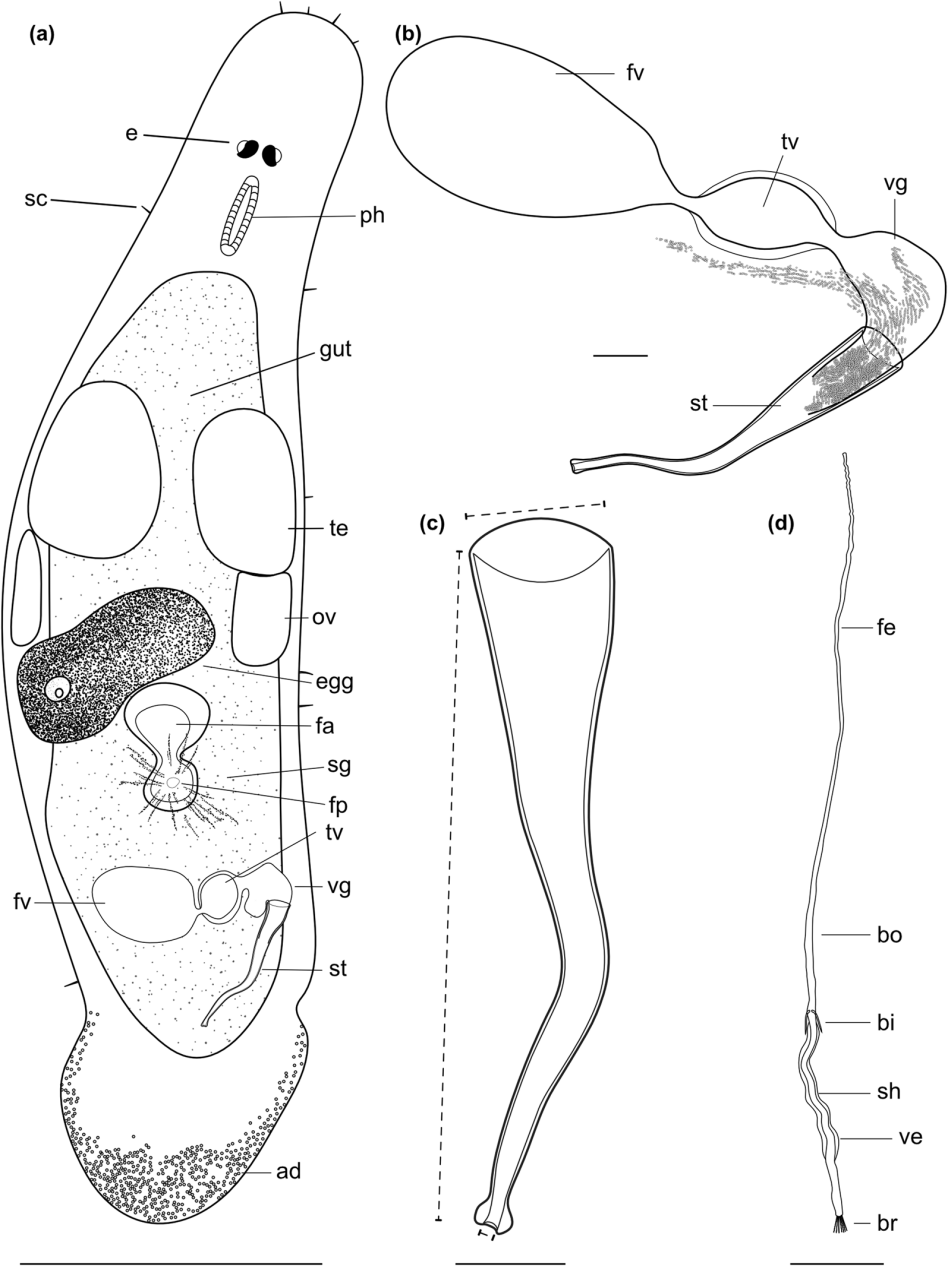
Line drawings of *Macrostomum schareri*. **a** Habitus (lightly squeezed), with sensory cilia (sc), eyes (e), pharynx (ph), testis (te), ovary (ov), female gonopore (fp), female antrum (fa), shell glands (sg), and adhesive glands (ad). **b** Male genital system with false seminal vesicle (fv), muscular true seminal vesicle (tv), *vesicula granulorum* (vg), and stylet (st). **c** Detailed drawing of the stylet (indicated are the measurements for the straight stylet length and the width of the proximal and distal stylet openings, while the segmented stylet length (in Table 3) corresponds to the average length of both sides of the stylet). **d** Mature sperm cell with feeler (fe), body (bo), bristles (bi), shaft (sh), and brush (br). Scale bars indicate 100 μm in (**a**) and 20 μm in (**b**–**d**)

**Fig. 14:**
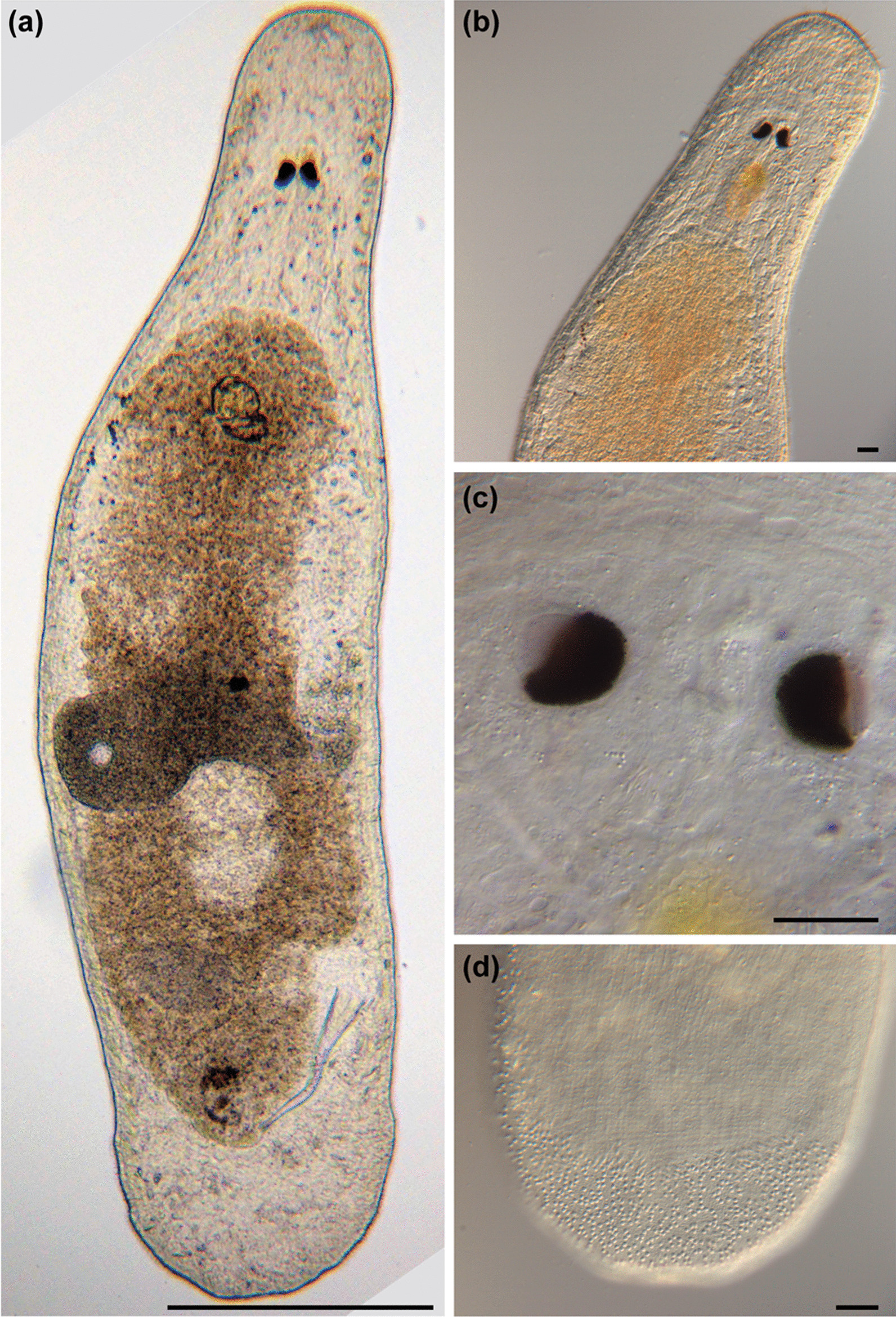
Micrographs of somatic structures of field-collected *Macrostomum schareri*. Brackets denote accessions of deposited specimens, with MTP LS 2688 being the HOLOTYPE. **a** Overview of an adult, lightly squeezed worm (MTP LS 2687). **b** Head region with neuropile, eyes, and pharynx; note the short sensory cilia on the rostrum (MTP LS 2687). **c** Large eyes with pigment cup and lens with rhammite gland secretions penetrating the neuropile (MTP LS 2687). **d** Tail region with dense adhesive glands (MTP LS 2687). Scale bars indicate 500 μm in (**a**) and 20 μm in (**b**–**d**)

**Fig. 15:**
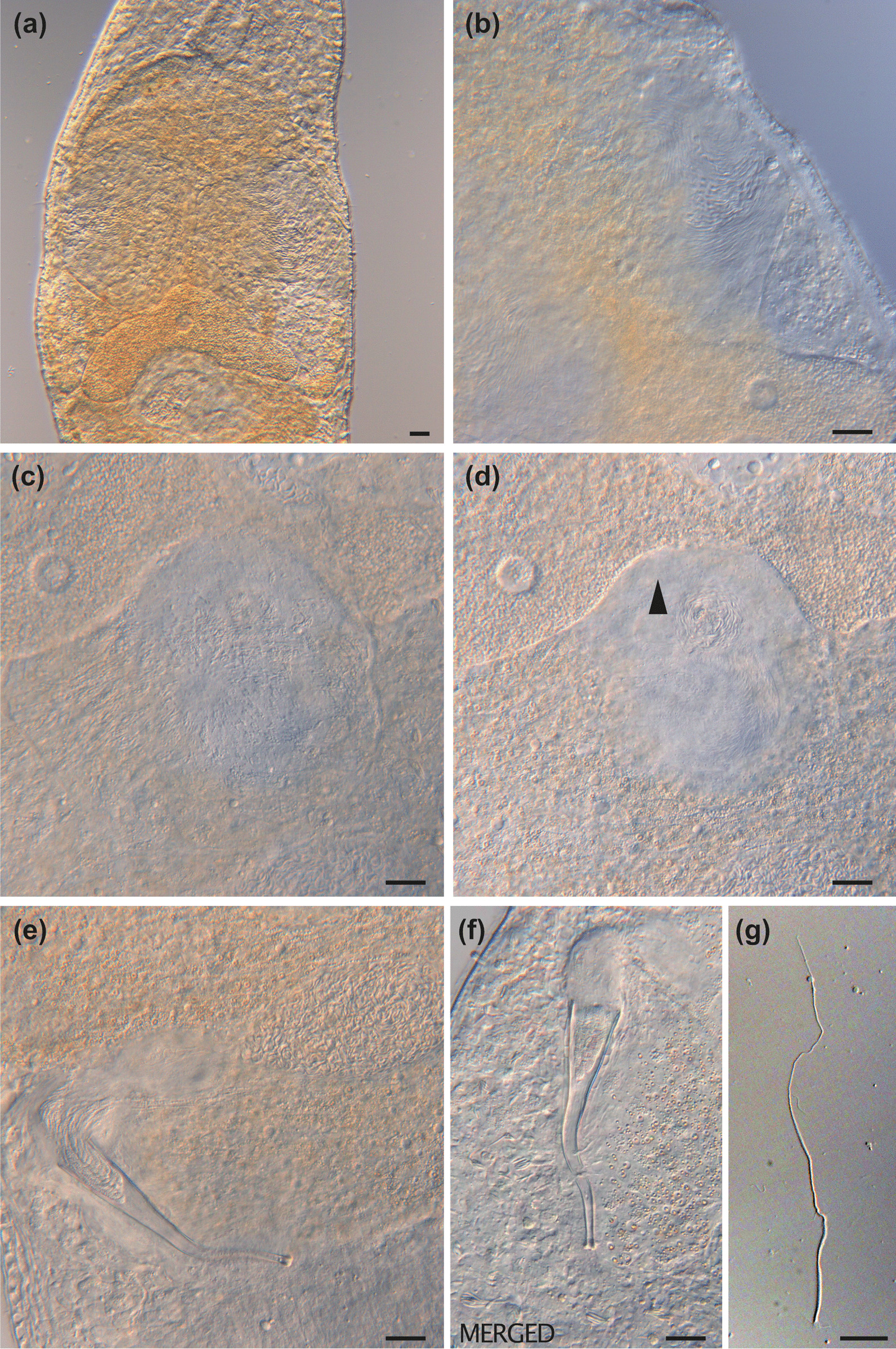
Micrographs of reproductive structures of field-collected *Macrostomum schareri*. Brackets denote accessions of deposited specimens, with MTP LS 2688 being the HOLOTYPE. **a** Overview of reproductive structures showing the testes, ovaries, an egg between the ovaries, and the female antrum filled with sperm (MTP LS 2700). **b** Detailed view of a ripe testis and ovary (MTP LS 2688). **c** Vagina with shell glands (MTP LS 2699). **d** Female antrum with a thick anterior epithelium (arrow) containing sperm (MTP LS 2699). **e** Overview of the male system with stylet, *vesicula granulorum*, and both seminal vesicles (MTP LS 2699). **f** Merged image stack of the stylet (MTP 2690). **g** Sperm with long anterior region, short bristles, and brush (MTP LS 2707). Scale bars indicate 20 μm

urn:lsid:zoobank.org:act:E92C6FC8-BB9C-49F5-86DE-65FBD6FA70AA (Figs. 13, 14 and 15; Table 3).

**Table 3:** Morphometric measurements of *Macrostomum schareri* and *Macrostomum gracilistylum*

Parameter	*M. schareri*				*M. gracilistylum*			
	Mean	SD	Range	N	Mean	SD	Range	N
Body L	918.4	152.5	633.8–1169.3	17	1083.0	131.4	801.9–1264.5	9
Body W	305.2	51.4	219.5–434.2	13	330.6	105.2	211.3–504.0	8
Eye ø	20.5	5.5	15.2–33.4	8	14.4	1.6	12.2–16.8	7
Testis L	158.1	23.8	137.1–221.9	11	166.5	27.6	114.1–193.3	7
Ovary L	99.3	18.0	75.7–142.9	11	88.9	20.5	61.4–115.8	7
Short sensory cilia L	12.8	3.9	8.2–22.4	13	10.2	2.2	5.6–12.5	8
Long sensory cilia L	20.8	5.0	11.6–28.1	8	23.4	5.1	18.4–33.1	8
Rhabdite L	6.7	1.6	5.3–9.4	5	10.1	1.2	9.1–11.9	4
Stylet traits								
Straight L	109.2	14.3	72–120.9	11	190.7	8.9	181.2–203.6	5
Segmented L	116.0	14.4	78.4–128.0	11	201.2	12.3	187.1–216.1	5
Proximal opening W	19.5	4.0	9.8–24.6	10	12.2	1.8	10.8–14.9	5
Distal opening W	3.0	0.9	2.1–5.2	11	1.8	0.2	1.4–2.1	5
Sperm traits								
Total L	168.4	10.0	159.2–179.0	4	70.6	1.9	67.9–72.4	4
Anterior L	121.4	7.6	114.3–129.0	4	52.0	1.4	50.6–53.5	4
Bristle L	3.8	1.0	2.8–5.1	4	5.3	1.0	4.3–6.7	4
Brush L	3.3	0.3	3.0–3.6	4	2.7	0.6	1.9–3.1	4

All length, width, and diameter measurements are in µm. L: length, W: width, ø: diameter

#### Material examined

Holotype: A digitally-documented specimen (MTP LS 2688), including 62 images and videos and a whole mount (Table 1), from the type locality collected on 1 September 2016 from the base of reeds in the surf zone of a rocky shore, Lake Tanganyika, Zambia. Paratypes: 17 digitally-documented specimens, including 587 images and videos, one partial *28 S rRNA* sequence, one whole-body transcriptome, and three whole mounts, comprising four specimens from the original sample and 13 specimens from a later sample of the type locality collected on 3 September 2016.

#### Etymology

The species is named in honor of Lukas Schärer for his contribution to *Macrostomum* research and involvement in collecting and describing this species. *M. schareri* has previously been referred to as *Macrostomum* sp. 74 or Mac074.

#### Diagnosis

Bottle-shaped *Macrostomum*, usually with narrower anterior than posterior region; body length between 633.8 and 1169.3 μm (see also Table 3); large eyes with laterally-facing pigment cups and large lenses; tail plate broad and distinct with a very large pad of adhesive organs; testes start at 20% BL and are large, sometimes touching at the midline; true seminal vesicle, false seminal vesicle, and *vesicula granulorum* lie on a straight line at 75% BL; gut extends far posterior of the male system; *vesicula granulorum* gland necks extend into the conical stylet base; stylet large; stylet shaft spirals with turns at 50%, 66%, and 90% of the length; protruding, symmetrical stylet thickenings at distal opening; ovaries small and directly posterior of the testes; female antrum with two chambers; first chamber ciliated, connecting via a muscular duct to a larger unciliated chamber.

Description (see also Table 3).

#### General morphology

Body bottle-shaped with a roundish rostrum and a broad tail plate (Figs. 13a and b and 14a). The rostrum is often active and moving laterally back and forth. The eyes are large with kidney-shaped pigment cups and large clear lenses (Fig. 14c). The pigment cups are facing laterally away from each other. Sensory cilia are short and homogeneously distributed along the body. The gut extends posteriorly past the male system terminating at 86% BL. The worm has a remarkable tail plate with numerous dense rows of adhesive organs arranged in a horseshoe-shaped pad (Fig. 14d).

#### Male system

**Fig. 16:**
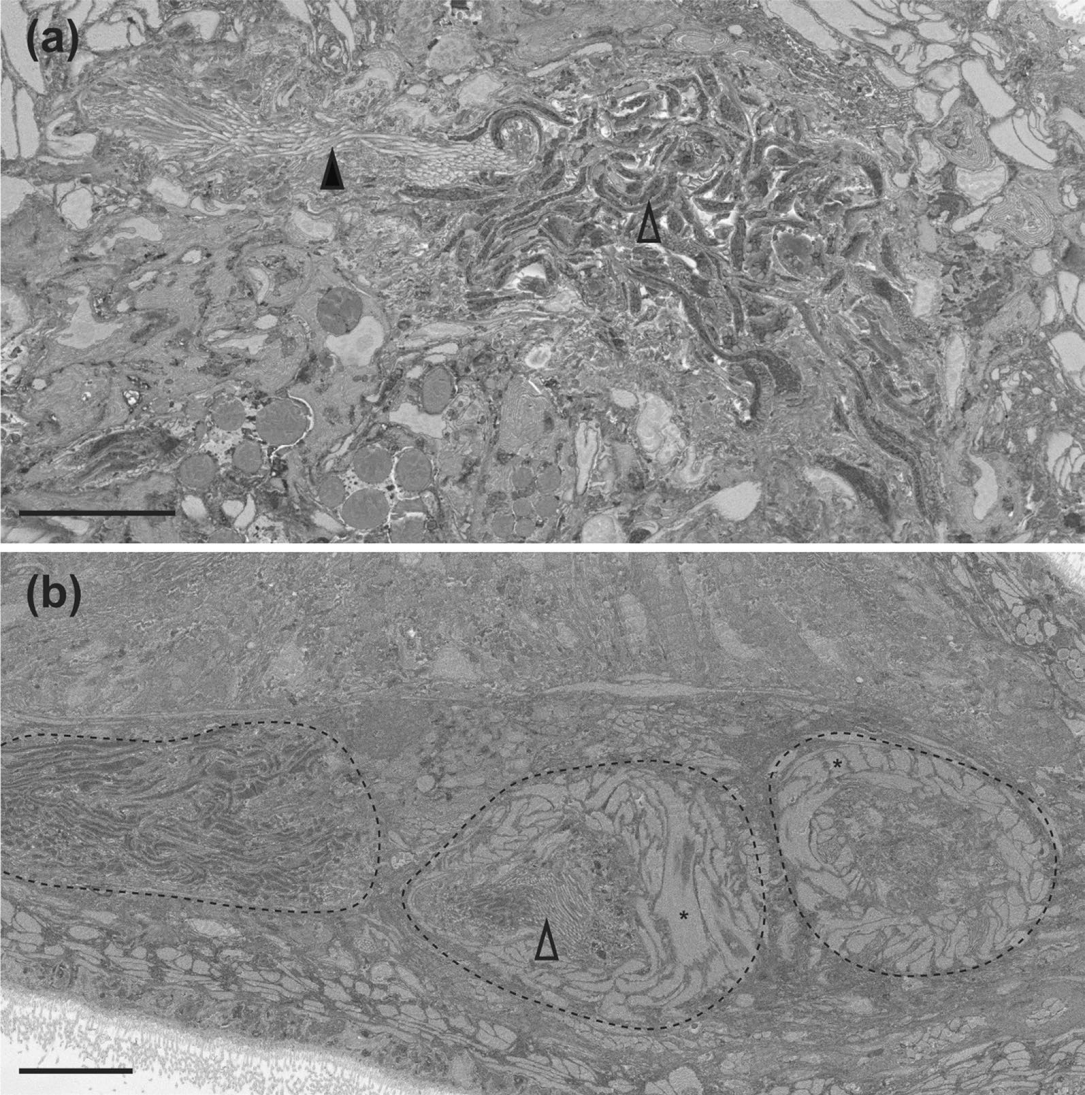
Serial block-face SEM images of male reproductive structures of *Macrostomum schareri*. **a** Sperm inside the false seminal vesicle (open arrowhead) and cilia lining the connection of the former to the true seminal vesicle (closed arrowhead) (MTP LS 2714). **b** Outlined are (from left to right) the false seminal vesicle, the true seminal vesicle with cilia (open arrowhead), and the *vesicula granulorum*. The asterisks indicate some of the strong musculature around the true seminal vesicle and the *vesicula granulorum* (MTP LS 2713). Scale bars indicate 10 μm

**Fig. 17:**
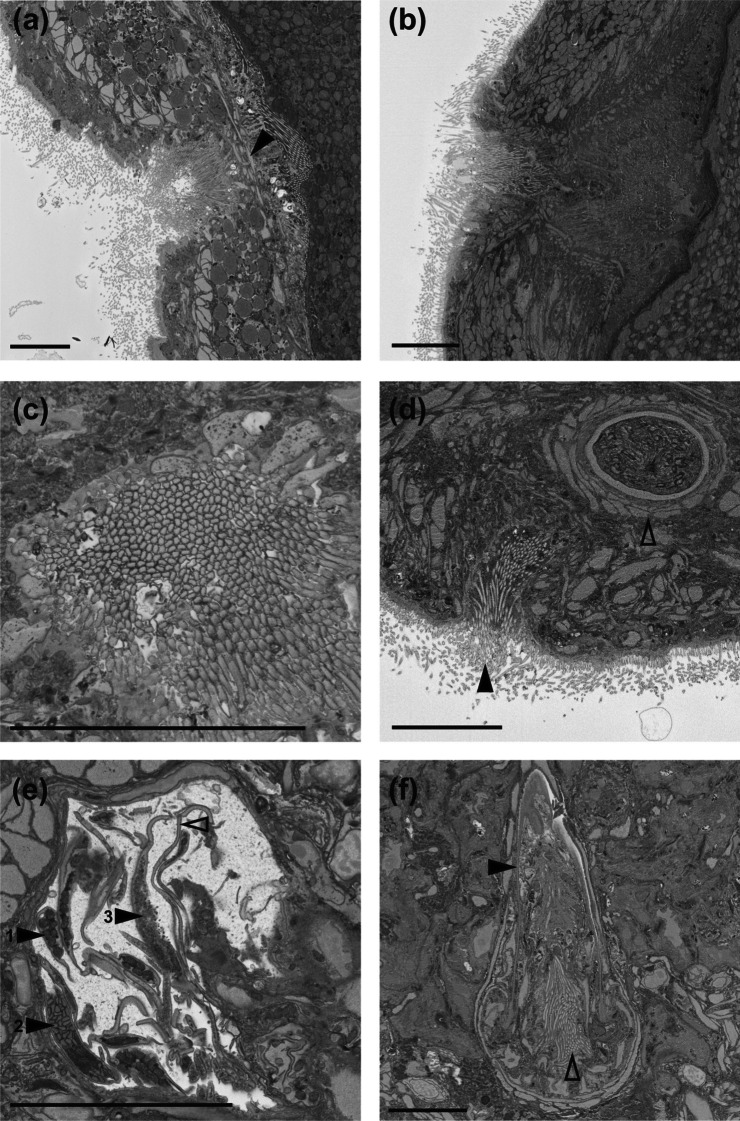
Serial block-face SEM images of reproductive structures of *Macrostomum schareri*. **a**, **b** Vagina with a clear muscular sphincter (closed arrowhead in a). **c** Section through the vagina wall showing dense cilia. **d** Details of the male system with the male gonopore (closed arrowhead) and the stylet with associated circular musculature (open arrowhead). **e** Vas deferens filled with sperm. Closed numbered arrowheads indicate mitochondria (1) and two types of vesicles (2, 3) within sperm. The open arrowhead indicates a very narrow region (~ 0.3 μm) of sperm. **f** Base of the stylet (black arrowhead) showing tissue of the *vesicula granulorum* with cilia (open arrowhead) penetrating it. The section from (**b**) is of specimen MTP LS 2714, all others are of MTP LS 2713. Scale bars indicate 10 μm

**Fig. 18:**
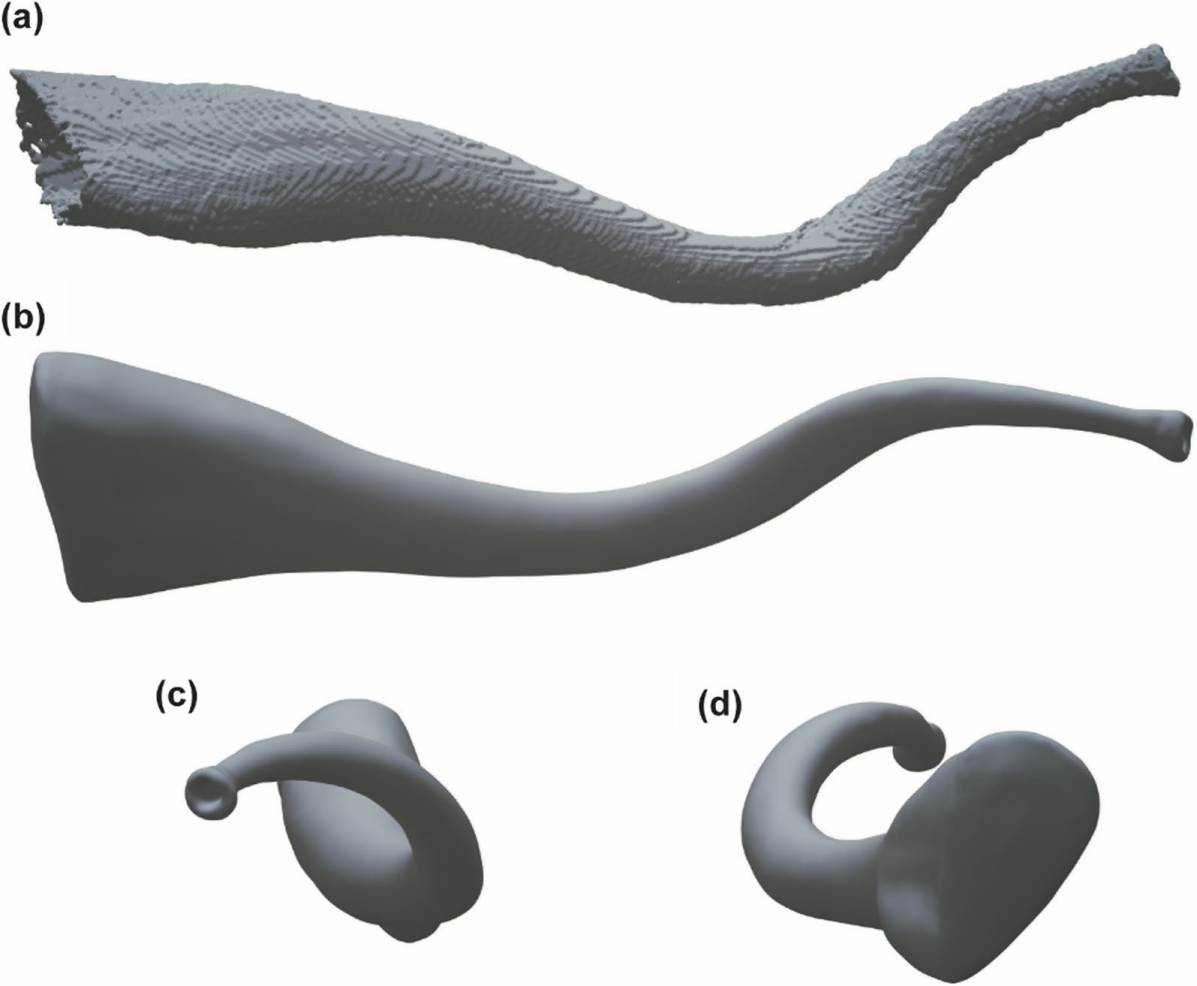
Images of the 3D reconstruction of the stylet of *Macrostomum schareri*. The reconstruction is based on 1026 serial sections of a Serial block-face Scanning Electron Microscope of specimen MTP LS 2713. **a** Reconstruction is based solely on the outlines of each section. (**b**–**d**) Images of the reconstruction after smoothing

The large testes start at 33% BL, extend to 50% BL, and are broad, often coming close together ventrally or even touching (Figs. 13a and 15b and d). The *vasa deferentia* extend posteriorly from the testes, leading to a thin-walled false seminal vesicle. The false seminal vesicle is connected via a ciliated duct (Fig. 16a) to the smaller, very muscular true seminal vesicle that lies on the same level (Fig. 16b). The true seminal vesicle is, in turn, connected to an elongated, muscular *vesicula granulorum* (Fig. 16b) that turns more than 90° to reach far into the conical stylet base (Figs. 13b and 17f). The stylet is unusually anterior, leaving a largely empty region in the tail plate (Figs. 13a and 14a). It has a broad conical funnel distally, gradually tapering towards the proximal opening. The shaft is a spiral with three turns at 50%, 66%, and 90% of its length (Figs. 13c, 15e and f and 18a–d). The distal opening has a symmetrical thickening that extends outwards. The ciliated male opening is quite large (Fig. 17d) and has a small field of glands associated with it, and is unusually far anterior at 82% BL. The anterior sperm length is thin, with a conspicuous thickening at 10% of its length. The sperm has short bristles at 72% of its length and a thin velum extending posteriorly from the bristles, terminating slightly before reaching the brush (Figs. 13d and 15g). Sperm contains at least two types of electron-dense bodies and regions densely packed with mitochondria (Fig. 17e).

#### Female system

The small, paired ovaries start at 45% BL, extend to 55% BL, and are directly posterior to the testes or overlapping the testes by up to 30% (Figs. 13a and 15a). There is often a central and surprisingly anterior developing egg, and the antrum is similarly anterior and frequently contains received sperm. Shell glands are unremarkable and extend radially from the female opening located at 62% BL (Fig. 15c). The vagina is heavily ciliated (Fig. 17a–c). The female antrum has two chambers. A muscular duct connects from the female opening into a thickened ciliated chamber, in turn leading anterior to a larger ciliated chamber. The anterior chamber has a thick epithelial wall that is particularly pronounced in the anterior region (Fig. 15d). Most specimens (10/18) had sperm in the antrum, usually located in the anterior chamber, and sperm was frequently embedded in the antrum wall (4/7 cases with sufficient image material).

#### Discussion

The spiral stylet of *M. schareri* is unique. Additionally, *M. schareri* possesses a distinctive combination of features, such as large eyes, elongated sperm, and a wide tail plate, setting it apart from all other species.

### *Macrostomum gracilistylum*, sp. nov.

**Fig. 19:**
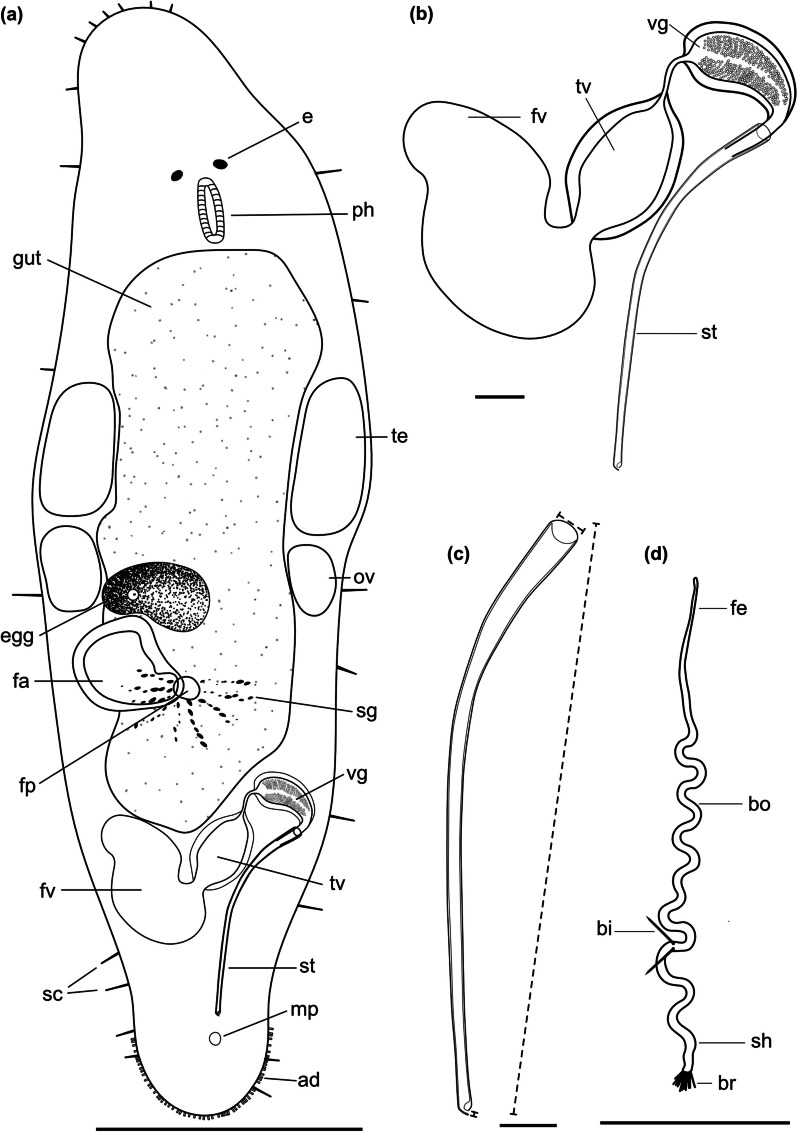
Line drawings of *Macrostomum gracilistylum*. **a** Habitus (viewed from dorsal and lightly squeezed), with sensory cilia (sc), eyes (e), pharynx (ph), testis (te), ovary (ov), female gonopore (fp), female antrum (fa), shell glands (sg), male gonopore (mp), and adhesive glands (ad). **b** Male genital system with false seminal vesicle (fv), muscular true seminal vesicle (tv), *vesicula granulorum* (vg), and stylet (st). **c** Detailed drawing of the stylet (indicated are the measurements for the straight stylet length and the width of the proximal and distal stylet openings, while the segmented stylet length (see Table 3) corresponds to the average length of both sides of the stylet). **d** Mature sperm cell with feeler (fe), body (bo), bristles (bi), shaft (sh), and brush (br). Note that it is difficult to distinguish the feeler and body region in this species. Scale bars indicate 100 μm in (**a**) and 20 μm in (**b**–**d**)

**Fig. 20:**
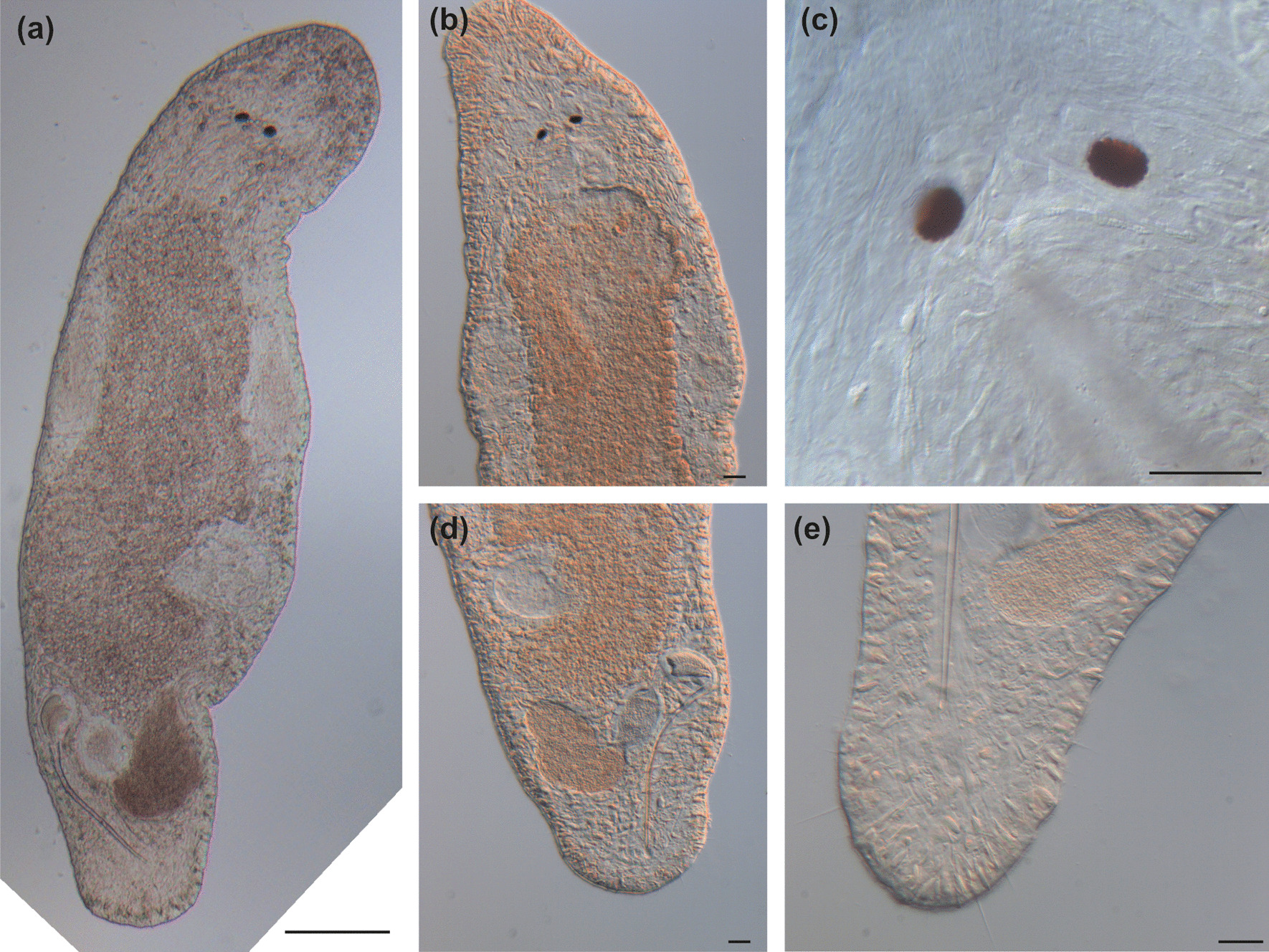
Micrographs of somatic structures of field-collected *Macrostomum gracilistylum*. Brackets denote accessions of deposited specimens, with MTP LS 3547 being the HOLOTYPE. **a** Overview of an adult, slightly squeezed worm (MTP LS 3549). **b** Anterior region showing the narrowing rostrum (MTP LS 3552). **c** Eyes and neuropile (MTP LS 3545). **d** Caudal region (MTP LS 3552). **e** Stretched tail plate with sensory cilia (MTP LS 3554). Scale bars indicate 100 μm in (**a**) and 20 μm in (**b**–**e**)

**Fig. 21:**
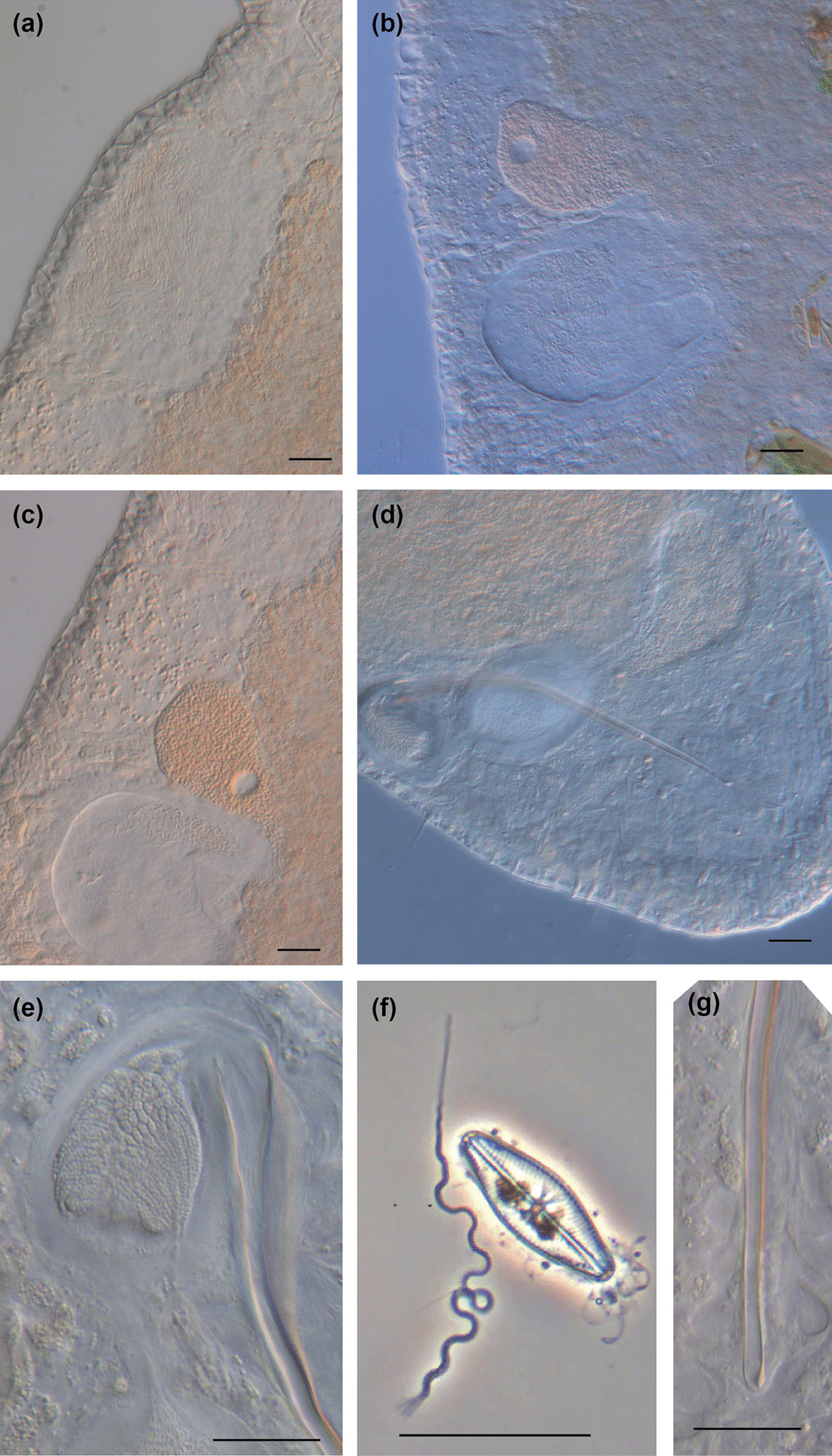
Micrographs of reproductive structures of field-collected *Macrostomum gracilistylum*. Brackets denote accessions of deposited specimens, with MTP LS 3547 being the HOLOTYPE. **a** Ripe testis (MTP LS 3551). **b** Ripe ovary, developing egg, and antrum with multiple sperm (MTP LS 3548). **c** Close-up of the ripe ovary, developing egg, and antrum with multiple sperm. Note the very similar arrangement to (**b**) (MTP LS 3551). **d** Overview of the male system with stylet, *vesicula granulorum*, and both seminal vesicles (MTP LS 3546). **e** *Vesicula granulorum* and base of stylet (MTP LS 3547). **f** Sperm next to a diatom, showing the sperm bristles and the terminal brush (MTP LS 3548). **g** Detail of the distal region of the stylet, showing the asymmetrical thickening and the remarkably narrow distal opening (MTP LS 3547). Scale bars indicate 20 μm

urn:lsid:zoobank.org:act:C9E1883A-4889-4CD4-B5A3-5857D7F4DA5C (Figs. 19, 20 and 21; Table 3).

#### Material examined

Holotype: a digitally-documented specimen (MTP LS 3546), including 112 images and videos, and a whole mount (Table 1), from the type locality collected in late September 2018 in clean coarse sand of approximately 2–3 mm grain size on a beach on the shore of Lake Malawi, Malawi. Paratypes: eight digitally-documented specimens, including 445 images and videos, two partial *28 S rRNA* sequences, and five whole mounts from the original sample at the type locality.

#### Etymology

From the Latin *gracilis*, meaning slender, referring to this species’ slender stylet. *M. gracilistylum* has previously been referred to as *Macrostomum* sp. 117 or Mac0117.

#### Diagnosis

*Macrostomum* with a small tail plate; body length between 801.9 and 1264.5 μm (see also Table 3); Eyes round and close together; testes larger than ovaries; false seminal vesicle without musculature and larger than true vesicle; true seminal vesicle with clear musculature, connected posteriorly to false seminal vesicle, stylet with slightly conical funnel-shaped base, turn of 30° at 30% of its length, leading to a slender, straight tube; distal stylet opening with a drop-shape thickening on the concave side and narrow subterminal opening; antrum displaced to the left side, ciliated, with a thick anterior epithelium; sperm width roughly constant, save for the most distal part; sperm with two bristles and a brush.

Description (see also Table 3).

#### General morphology

A *Macrostomum* with a small tail plate (Figs. 19a and 20a and b). The rostrum is short and has prominent rhabdites. The eyes are round and close together, with small lenses (Fig. 20c). Short and sensory cilia are distributed all along the body (Fig. 20e). The tail plate has several rows of adhesive organs. The gut extends posteriorly until the false seminal vesicle. Since the *vesicula granulorum* lies anterior to the seminal vesicles, the gut has an asymmetric shape extending further on the left side (Fig. 20d).

#### Male system

The testes are larger than the ovaries, starting at 32% BL and extending to 49% BL (Fig. 21a). The *vasa deferentia* join in a large thin-walled false seminal, which can be 2–3 times larger than the true seminal vesicle. The true seminal vesicle has a thick muscular wall and a long ductus intervesicularis connecting it to the muscular *vesicula granulorum* (Fig. 19b). The false seminal vesicle connects to the posterior end of the true seminal vesicle, and the true vesicle is usually more posterior than the *vesicula granulorum*. Consequently, the gland necks from the *vesicula granulorum* make a sharp 90° turn to protrude only slightly into the base of the stylet (Fig. 21e). The stylet is a long narrow tube with a somewhat wider conical base with a 30° turn at 30% of its length, leading into a slender, straight tube with almost the same diameter along its length. The distal opening is characteristic with a drop shape thickening on the concave side and a thin wall covering the other side (Figs. 19c and 21g). Because of this, the opening is very narrow and subterminal. The sperm is slender and has two bristles and a brush (Figs. 19d and 21f).

#### Female system

The ovaries are smaller than the testes, starting at 49% BL and extending to 57% BL (Fig. 21b,c). The female pore lies centrally at 61% BL, is ciliated, and is surrounded by prominent shell glands (Fig. 19a). The antrum is displaced to the left and consists of one large ciliated chamber with a pronounced cellular valve. Sperm is often observed inside the antrum embedded in the anterior right side of the chamber, where presumably the mature eggs enter. The anterior and the right anterior region have a thick epithelium and appear to have some structure with several folds at the location where sperm is embedded (Fig. 21b,c). Most specimens (7/9) had sperm in the antrum. Sperm was clearly embedded in the antrum wall in five specimens.

#### Discussion

Among the African freshwater species, *M. gracilistylum* has some resemblance to *Macrostomum salemensis* Belatgi & El-Said, 2003, and *M. coxi* Young, 1976. The stylet of *M. gracilistylum* shares similarities with *M. salemensis* since both stylet are curved and have a subterminal opening. However, the stylet of *M. salemensis* is 20 μm shorter and has a broader base. Furthermore, the subterminal opening is different, with a thickening on one side in *M. gracilistylum* that is absent in *M. salemensis*. *M. gracilistylum* resembles *M. coxi* in stylet length (200 μm) and the long duct between the true seminal vesicle and *vesicula granulorum*. Young draws several versions of the distal opening, one of which (his Fig. 1C) resembles *M. gracilistylum*. However, the species are distinct because *M. coxi* does not have a subterminal stylet opening, the stylet base is wider, and the female opening is more posterior. Among all other *Macrostomum* species, *M. gracilistylum* bares some similarities with *Macrostomum lutheri* Papi, 1951. Both species have a stylet with a similar curve and a subterminal opening with a thickening on the concave side. They might also be of similar length since Papi [56] says that the longest stylet found was 210 μm, but curiously there seems to be a wide range in length, with the one drawn in his Fig. 6 only measuring 86 μm. However, they differ clearly in the shape of the narrow proximal opening, which is 12.2 μm in *M. gracilistylum* but funnel-shaped and 50 μm wide in *M. lutheri*.

### Nomenclatural acts

The variety *M. tuba* var. *verbekei* Marcus & Marcus, 1957 is raised to species level and designated *Macrostomum verbekei* Marcus & Marcus, 1957.

## Discussion

### *Macrostomum* radiation

The Tanganyika clade is monophyletic and likely diverged within a short period. In the previously published phylogeny, the Tanganyika clade has a short branch length. Indeed, the largest within-clade distance is shorter than the separation of the model species *M. lignano* and its sister *M. janickei* Schärer, 2019 with the next closest known relative *M. cliftonense* (Additional file 2: Fig. S2 A in [13]). Although I can compare these species without a calibrated phylogeny, it is not possible to accurately date the tree and, therefore, the age of the Tanganyika clade. If I had such a phylogeny, I could determine if this clade is younger than the lake or if it represents an older radiation that predates the lake formation. Unfortunately, no *Macrostomum* fossils exist, and the generally low endemicity across the genus prevents geographic calibration of phylogenetic nodes. However, the apparent endemism within the Tanganyika clade is unusual and in itself suggests an interlacostral origin. But note that the sampling was not extensive enough to firmly conclude that the Tanganyika clade is truly endemic to the Lake.

The Tanganyika clade displays a high degree of morphological diversity. The comparative analysis showed that sperm in this clade is characterized by a novel morphology, including simultaneous changes in sperm length, sperm ratio, and bristles length (Fig. 3). Species in the Tanganyika clade also have a great diversity of male stylets and female antra [13, 19]. Additionally, two species in the clade have lost their sperm bristles. Other unusual characteristics, not related to sexual reproduction, have also emerged, particularly in *M. schareri*, which has unusually large pigment cup eyes with large lenses (Fig. 14c) and a broad tail plate with many adhesive organs (Fig. 14d), potentially related to this species living on reeds that are exposed to intense wave action.

The Tanganyika clade shows remarkable habitat diversity, likely due to the greater number of habitats available in Lake Tanganyika compared to surrounding rivers. Interestingly, several species were exclusively found in only one type of habitat. For example, some species were found in the sand around snail shells, which are maintained by cichlids for breeding and shelter [57] and are, therefore, a special habitat that is only available in Lake Tanganyika. Another species was found in a sponge, which is the first known record of such a habitat for *Macrostomum*. Given the remarkable sponge diversity in Lake Tanganyika [58], many more *Macrostomum* species may be specific to different sponge species. Importantly, even though species appeared to prefer particularly habitats, these habitats were not clustered phylogenetically in the Tanganyika clade, suggesting that different niches have been filled multiple times independently.

It is important to note that my assessment of sampling habitats is superficial, as the sampling was not systematic enough to draw firm conclusions about habitat specificity, and more careful investigations will likely uncover many more differences in habitats. For example, the category of a “sand habitat” does not include depth or grain size, but it is well documented that these factors have a significant influence on meiofauna, including *Macrostomum* species [59]. Indeed, the African Great Lakes may contain a large variety of interstitial niches for meiofaunal organisms such as *Macrostomum*. Similarly, I have combined all types of water plants and even dissolving wood into the “vegetation habitat” type for simplicity, but some species were only collected from specific plant species.

In summary, the Tanganyika clade meets the broad criteria of an adaptive radiation, showing rapid speciation and ecological and morphological diversification. At this stage in its study, I am unable to conclude that it constitutes an adaptive radiation according to the criteria of Schluter (2000). I provided evidence for rapid speciation and common descent but could not assess trait utility and phenotype-environment correlation (Table 4).

**Table 4:** Evidence for an adaptive radiation in the Tanganyika clade following the criteria of Schluter (2000)

Criteria	Status in the Tanganyika Clade
Common descent	Yes
Rapid speciation	Yes
Phenotype-environment correlation	Unclear: Diversification of habitat and morphology.
Trait utility	Unclear: Candidate traits for reproduction and ecology.

Despite the promising evidence of an adaptive radiation within the Tanganyika clade, it is still unclear if the radiation occurred within Lake Tanganyika. The data are consistent with the rapid speciation of *Macrostomum* in Lake Tanganyika and subsequent expansion into Lake Malawi, but the sampling is limited and does not allow firm conclusions. If members of the Tanganyika clade were found in other regional water bodies, this would challenge the hypothesis. Surveys of some habitats near Lake Tanganyika revealed several new species. Therefore, adequate habitats exist close to the lake. However, these species do not cluster phylogenetically with the Tanganyika clade, in agreement with the interlacostral speciation hypothesis. Additional sampling of Lake Malawi is needed to test if other species there cluster with *M. gracilistylum* and are, therefore, also nested in the Tanganyika clade or if they are more distantly related to them.

Within Lake Tanganyika, more than 40 species of Monogenean flatworms of the genus *Cichlidogyrus* parasitizing cichlids have been described [60]. The available molecular data suggests that the Monogenean species show high host specificity and that their speciation is likely primarily host dependent [61, 62] and thus dissimilar to the *Macrostomum* radiation. Furthermore, only 15 species have been included in a recent phylogeny, so it remains unclear if the entire genus is monophyletic and if it co-radiated with the cichlid host [62, 63]. To my knowledge, the most similar radiation has potentially occurred in Lake Baikal. Lake Baikal contains more than 70 endemic flatworms from 11 families, likely resulting from frequent colonization [64]. The most well-documented radiations occurred among the Dendrocoelidae planarians, giving rise to at least 21 endemic species in the Archicotylus species flock and at least 12 endemic species in the Bdellocephala species flock [64]. But note that molecular data for the Baikal Bdellocephala and Archicotylus are sparse, so these radiations could also consist of several independent invasions. The *Macrostomum* radiation is, therefore, comparable to the diversity in both the *Cichlidogyrus* and Lake Baikal radiations.

### Generalist ancestors

The progression of a radiation is often assumed to involve initial colonizing species that are generalists, which then become more specialized as they adapt to available habitats and speciate [7]. The phylogenetic distribution supports this scenario. The sister clade contains five species from Zambia, two in Lake Tanganyika, and three from other water bodies. The other members of the clade are mostly larger (2–3 mm body length) species commonly found in modified environments. *M. acus* Wang, 2005 and *M. obtusa* Wang, 2005 were found in water reservoirs in Shenzhen, China [65], while *M. tuba* and *M. quiritium* have a global distribution and are frequently found in ponds and botanical gardens [13, 66–69]. *M. poznaniense* is found in botanical gardens [70] and in various aquaria, particularly those for *Danio rerio* (pers. obs.). *M. gieysztori* has been collected from canals in Spain and Italy [13, 71], and its sister species are found in springs in Tunisia [13]. This global distribution suggests that the ancestor of this clade was efficient at colonizing new habitats, consistent with the generalist ancestor hypothesis.

### Functional hypotheses for anterior sperm elongation

The changes in sperm traits within the Tanganyika clade suggest a functional adaptation, possibly in the context of sexual conflict. Members of reciprocally mating *Macrostomum* species flatworms will frequently perform a post-copulatory “suck behavior” in an apparent attempt to remove the received ejaculate [15, 21]. Indeed, at least in one species, it has been observed that the behavior removes ejaculate (pers. obs.). Although, the function of the suck behavior has not completely been elucidated it likely arises due to conflict over mating roles (reviewed in [72–74]). During the suck behavior, both sperm feeler and sperm bristles may function as a persistence trait countering sperm removal (Fig. 1b & c) [21]. Reciprocal copulation and the suck behavior have been observed in several members of the Tanganyika clade (pers. obs.). Therefore, it is reasonable to discuss sperm morphology in relation to these behaviors.

Elongated sperm and an increased sperm ratio might allow deeper embedding into the tissue, enhancing resistance to the suck behavior (Fig. 1d). Of the species described here, sperm was seen embedded in the antrum wall in *M. pellitum*, *M. schareri*, and *M. gracilistylum*. However, in six observations of received sperm in *M. longispermatum*, I never observed the sperm embedded. Interestingly, the suck behavior was also not observed in *M. longispermatum* (pers. obs.), and this species has the longest sperm of any known *Macrostomum*. Thus, the species’ sperm may effectively anchor into the antrum, rendering the suck behavior ineffective and leading to its loss. However, the limited observations warrant further research for more conclusive findings.

The comparative analysis revealed that in the Tanganyika clade, sperm elongation is accompanied by a decrease in bristle length. This supports the functional hypothesis that the feeler provides resistance against the suck behavior and, therefore, bristles may no longer result in significant fitness benefits. Members of the Tanganyika clade could be in the process of losing the bristles. Indeed, previous work shows that bristles are frequently lost across the genus [19] and suggests these losses can occur due to other factors besides the origin of hypodermic insemination. Brand et al. [19] documented two species without sperm bristles but with a blunt stylet and a complex female antrum (*M*. sp. 82 from Australia and *M*. sp. 68 in the Tanganyika clade), which is the typical morphology for reciprocal copulation [19]. Therefore, *M*. sp. 68 may represent an advanced stage of the bristle reduction observed across the Tanganyika clade. Also note that *M*. sp. 64, the earliest branching member of the Tanganyika clade, has simple sperm without bristles, typical for species mating through hypodermic insemination (Fig. 2c) [19].

Alternatively, to the outlined scenario, sperm elongation could also facilitate sperm-egg interaction (Fig. 1e). Perhaps, stimulating egg maturation or allowing accelerated sperm-egg fusion. Both mechanisms could be beneficial in male-male competition by shortening the interval between mating and fertilization and therefore reducing the risk of sperm displacement by a competitor.

## Conclusions

Given the apparent abundance and diversity of *Macrostomum* in Lake Tanganyika and the presence of at least one species in Lake Malawi, it is worthwhile to focus more research on this diversity in these lakes. Additionally, other African Great Lakes may harbor significant *Macrostomum* diversity, potentially representing a hotspot of speciation similar to the other well-known radiations. It can then be tested if geographic patterns of diversity found in other systems are replicated within the genus. For example, past research has shown that species diversity in the African Great Lakes is related to the time since the last major ecological disturbance of a lake [75]. Additionally, further studies can investigate the role of intrinsic and extrinsic factors in driving the radiation, as well as the extent of niche sharing and coevolution within the clade. Overall, the *Macrostomum* radiation is a promising system for studying how sexual selection drives speciation on a macroevolutionary scale.

### Supplementary Information

**Additional file 1**. Supplementary tables detailing sperm measurements and the comparative analyses.

**Additional file 2**. Supplementary information on serial block-face fixation protocols and supplementary figures of the comparative analyses.

## Data Availability

The datasets generated during or analysed during the current study are available in the zenodo repositories, https://zenodo.org/record/2602479, https://zenodo.org/record/5656981, https://zenodo.org/record/6353143, and https://zenodo.org/record/6353143. The script used for the comparative analysis is available at https://github.com/Jeremias-Brand/FIZ_analysis.
