# Correction: Support for a radiation of free-living flatworms in the African Great Lakes region and the description of five new *Macrostomum* species

**DOI:** 10.1186/s12983-024-00526-2

**Published:** 2024-03-05

**Authors:** Jeremias N. Brand

**Affiliations:** 1 Department of Environmental Sciences, Zoological Institute, University of Basel, Vesalgasse 1, Basel, 4051 Switzerland; 2 Department of Tissue Dynamics and Regeneration, Max Planck Institute for Multidisciplinary Science, Am Fassberg 11, 37077 Göttingen, Germany

**Correction: Frontiers in Zoology (2023) 20:31** 10.1186/s12983-023-00509-9

Following publication of the original article [1], the authors reported an error in the spelling of a species name.

Based on the taxonomic code of zoology (https://code.iczn.org/formation-and-treatment-of-names/article-32-original-spellings), the species name “Macrostomum schäreri” in the article should be corrected to “Macrostomum schareri”.

The original article [1] has been updated.
