# Supplementary material for: Support for a radiation of free-living flatworms in the African Great Lakes region and the description of five new Macrostomum species
Source: Front Zool. 2023 Sep 5;20:31. doi: 10.1186/s12983-023-00509-9 (PMC10478486; doi:10.1186/s12983-023-00509-9)
Supplement: Supplementary file 2 — Additional file 2. Supplementary information on serial block-face fixation protocols and supplementary figures of the comparative analyses. [file 12983_2023_509_MOESM2_ESM.pdf]

1 Fixation for Serial Block Face SEM

Solutions

Sorensen's stock	Sorensen's Stock A<br>2.76 g $NaH_2PO_4 \bullet H_2O$ (Sigma Aldrich) in 100 mL ddH_2O<br>Sorensen's Stock B<br>2.84 g $NaHPO_4$ (Sigma Aldrich) in 100 mL ddH_2O<br>0.2 M Sorensen's phosphate stock<br>combine 28 mLl Stock A and 72 mL Stock B
Trump's phosphate	0.1 M Sorensen's stock, pH 7.2<br>0.1 mM $CaCl_2$<br>4% Paraformaldehyde (EMS)<br>1% Glutaraldehyde (EMS 16300)<br>10% Sucrose
Osmium solution A	Mix 2 % aqueous OsO_4 (EMS 19100) and 3% Potassium ferrocyanide (Sigma Aldrich 60280), 4 mM $CaCl_2$ in 0.3 M Cacodylatebuffer (Sigma Aldrich) prepare just before use.
Osmium solution B	2 % aqueous OsO_4 (EMS) in ddH_2O
UA solution	1% aqueous uranyl acetate (EMS 22400) in ddH_2O
TCH solution	1% thiocarbohydrazide (Sigma Aldrich 88535), incubated at 60°C for 1 h, swirl every 10 min, let cool to RT, filter through 22 µm syringe filter
Walton's lead aspartate	0.066 g lead nitrate (EMS 17900) in 10 mL 0.03 M aspartic acid (Sigma Aldrich 1043819), adjust pH to 5.5 using 1 M KOH (Sigma Aldrich), incubate at 60°C for 30 min, filter through 22 µm syringe filter before use
Durcupan	11.4 g Part A (Sigma Aldrich 44611), 10 g Part B (Sigma Aldrich 44612), 0.64 g BDMA (Serva 14835), 0.05–0.1g (err toward 0.05), Part D (Sigma Aldrich Sigma 44614)

Protocol

Fixation

- Relax animals using ~0.1% Phenoxypropanol — 5 min
- Fix with Trump's phosphate — 1 h (4°C)

Staining day 1

- Wash in 0.1 M Cacodylate buffer **without** sucrose — 5 × 3 min
- Prepare 2 **glass pipettes** with a measuring line at an equal height
- Osmium solution A: — 1 h (4°C)
  Remove the Cacodylate buffer, then add Osmium and then Ferrocyanide to the well, using the glass pipettes
- Start preparing the TCH solution:
  Prepare the solution and incubate at 60°C for 1 h. Agitate solution regularly.
- Wash in ddH_2O — 5 × 3 min
- Add TCH solution — 20 min (RT)
- Wash in ddH_2O — 5 × 3 min
- Osmium solution B: — 30 min (RT)
  Use glass pipette!

- Wash in ddH_2O 5 × 3 min
- UA solution ON (4°C)

Staining day 2

• Prepare Walton's lead aspartate solution and place it in the oven	30 min (60°C)
• Wash in ddH_2O	5 × 3 min
• Pre-heat the samples to 60°C	
• Walton's lead aspartate (**maximal time**): Filter (22 µm) solution directly into dishes	30 min (60°C)
• Prepare the EtOH solutions	
• Wash in ddH_2O	5 × 3 min
• Wash in 25%, 50%, 70%, 90% EtOH	4 × 10 min (4°C)
• Start preparing the resin	
• Wash in 100% EtOH	3 × 10 min (4°C)
• Wash in 100% acetone	10 min (4°C)
• Infiltrate in acetone with 1 drop of Durcupan	ON (4°C)

Infiltration

• Infiltrate in acetone with 2 drops of Durcupan	ON (4°C)
• Infiltrate in 10% Durcupan in acetone	ON (4°C)
• Infiltrate in 25% Durcupan in acetone	ON (4°C)
• Infiltrate in 50% Durcupan in acetone	ON (4°C)
• Infiltrate in 70% Durcupan in acetone	ON (4°C)
• Transfer to a small container	
• Infiltrate in 90% Durcupan in acetone	ON (4°C)
• Infiltrate in 100% Durcupan	ON (RT)
• Infiltrate again in 100% Durcupan	3 × 1 h (RT)
• Mount specimen in an appropriate container	
• Harden resin in oven	48 h (60°C)

2 Supplementary Figures

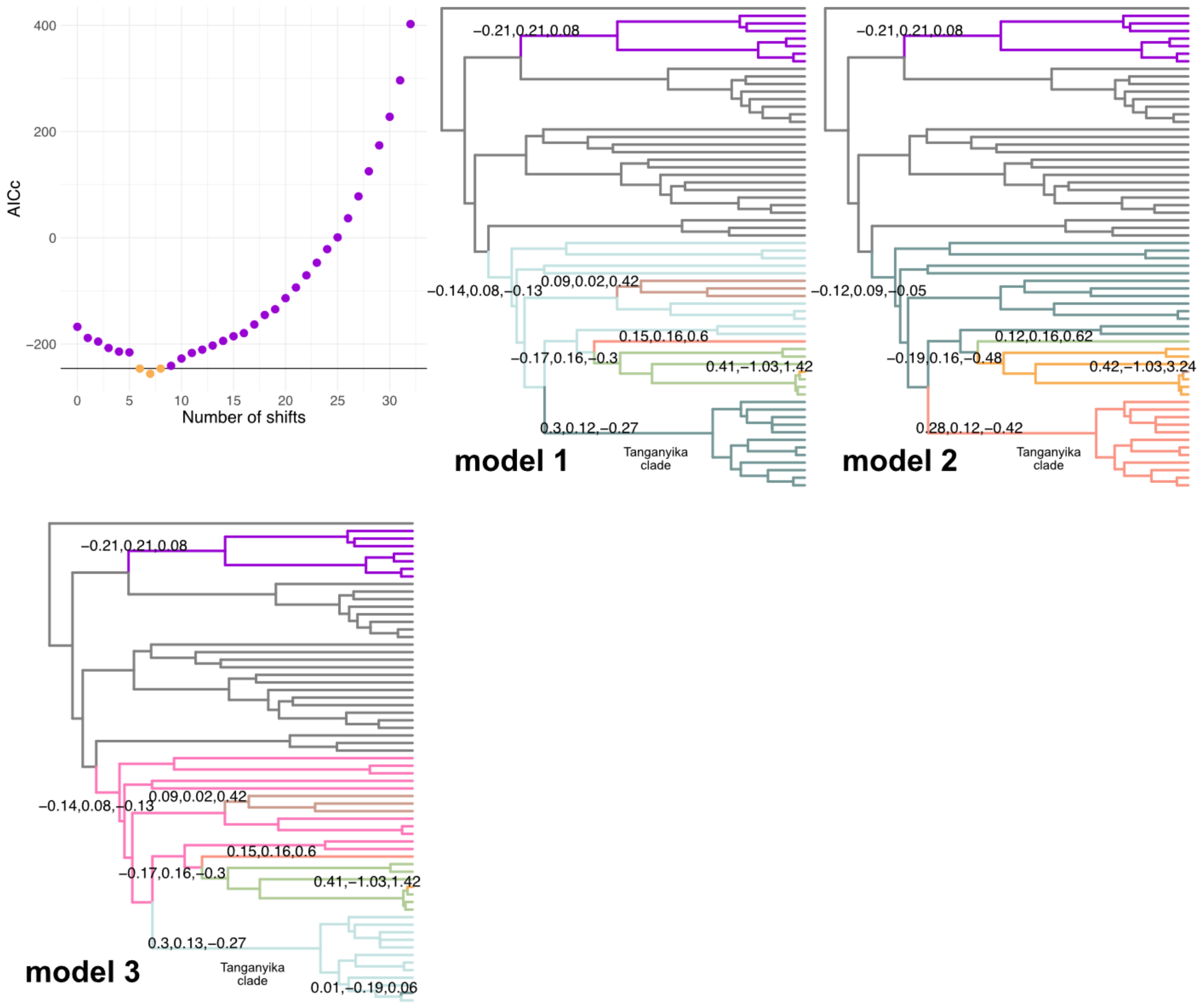

Figure S1 AICc profile and inferred Ornstein-Uhlenbeck shifts for the multivariate *l1ou* model including sperm length, sperm ratio, and bristle length. Shift configurations that differred by 10 AICc or less from the best configuration (model 1) are highlighted in yellow in the first plot and vizualized. Values at the shift locations indicate the magnitude and direction of the shift.

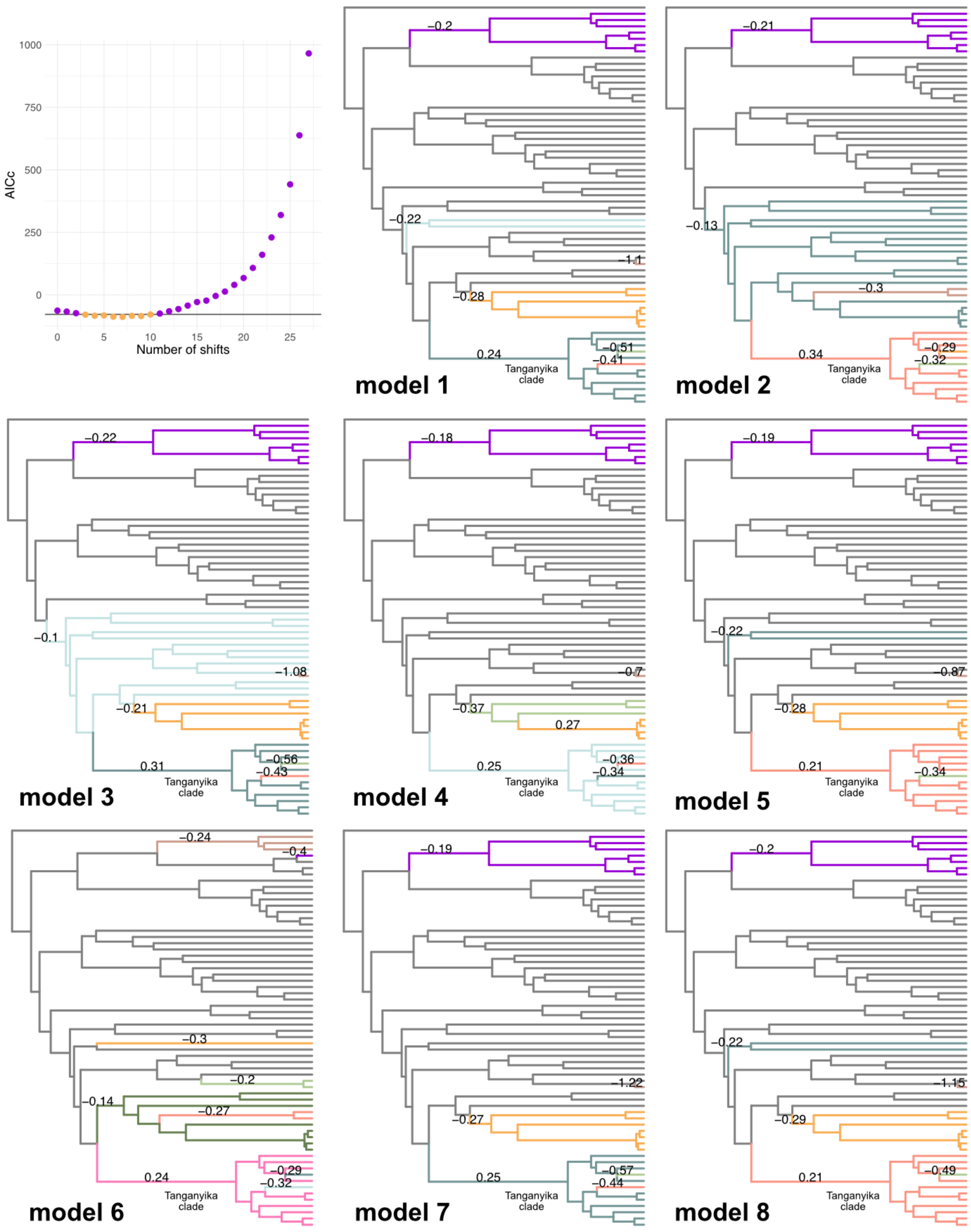

Figure S2 AICc profile and inferred Ornstein-Uhlenbeck shifts for the *l1ou* model including total sperm length. Shift configurations that differed by 10 AICc or less from the best configuration (model 1) are highlighted in yellow in the first plot and vizualized. Values at the shift locations indicate the magnitude and direction of the shift.

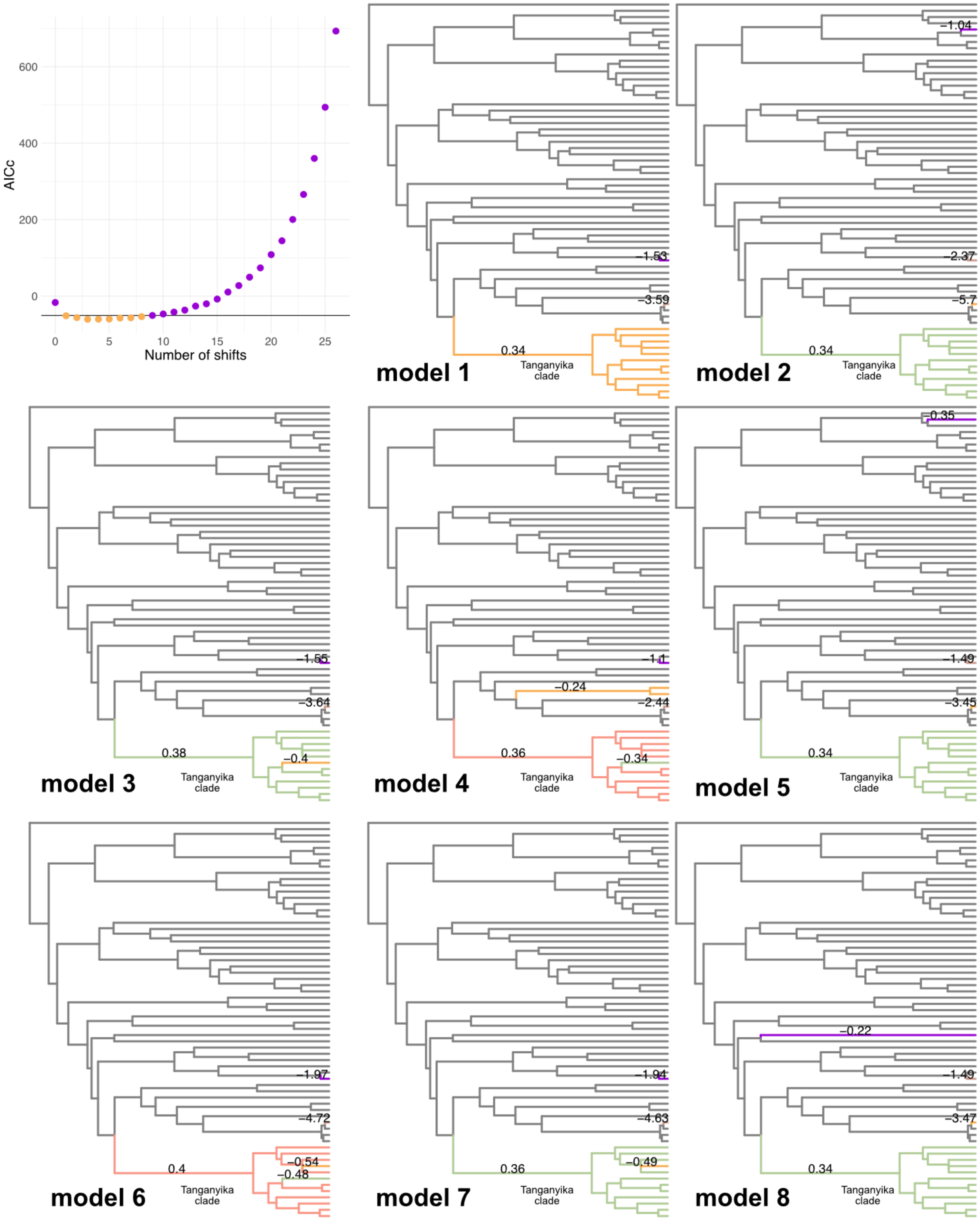

Figure S3 AICc profile and inferred Ornstein-Uhlenbeck shifts for the *l1ou* model including anteriro sperm length. Shift configurations that differed by 10 AICc or less from the best configuration (model 1) are highlighted in yellow in the first plot and vizualized. Values at the shift locations indicate the magnitude and direction of the shift.

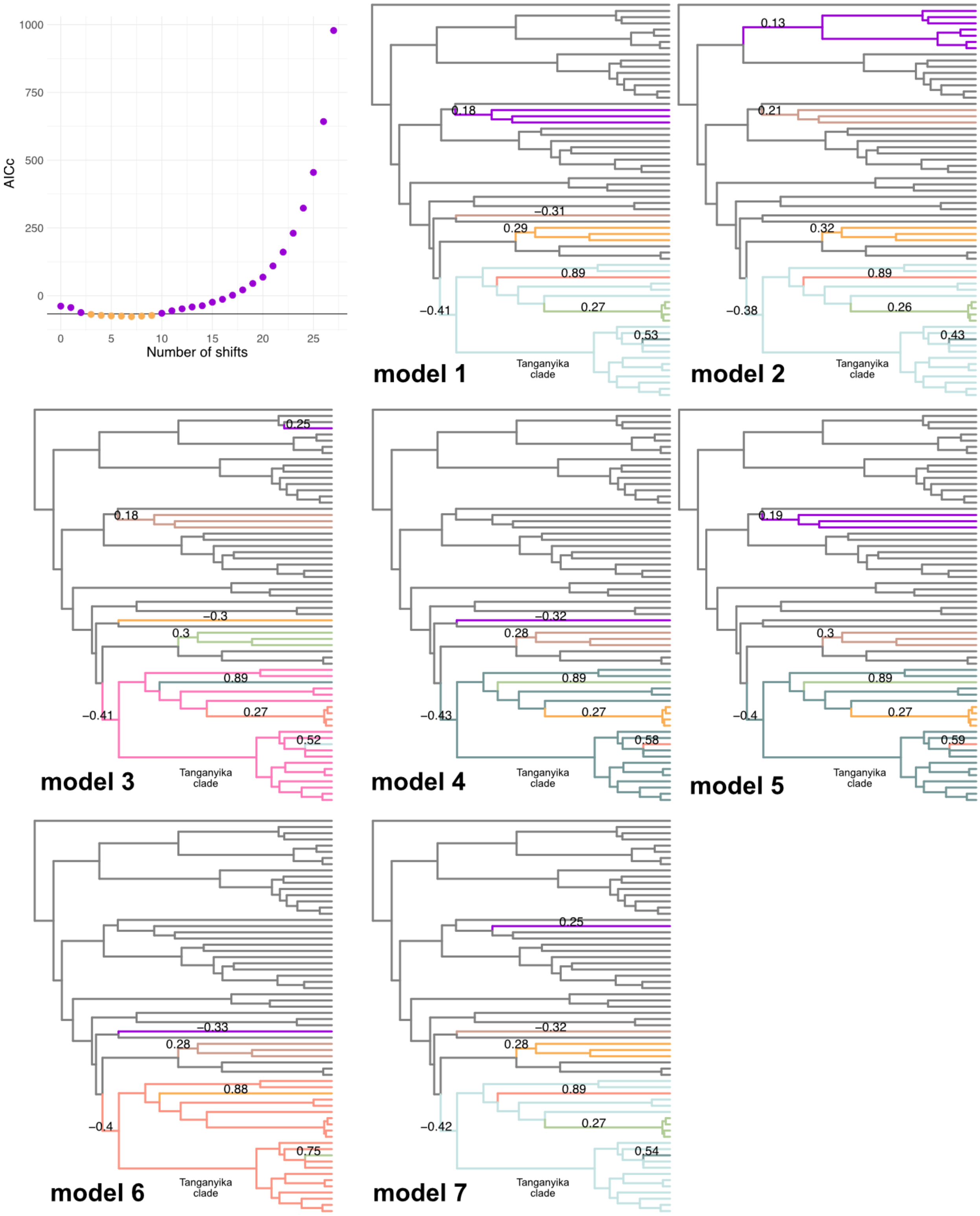

Figure S4 AICc profile and inferred Ornstein-Uhlenbeck shifts for the *l1ou* model including bristle length. Shift configurations that differed by 10 AICc or less from the best configuration (model 1) are highlighted in yellow in the first plot and vizualized. Values at the shift locations indicate the magnitude and direction of the shift.

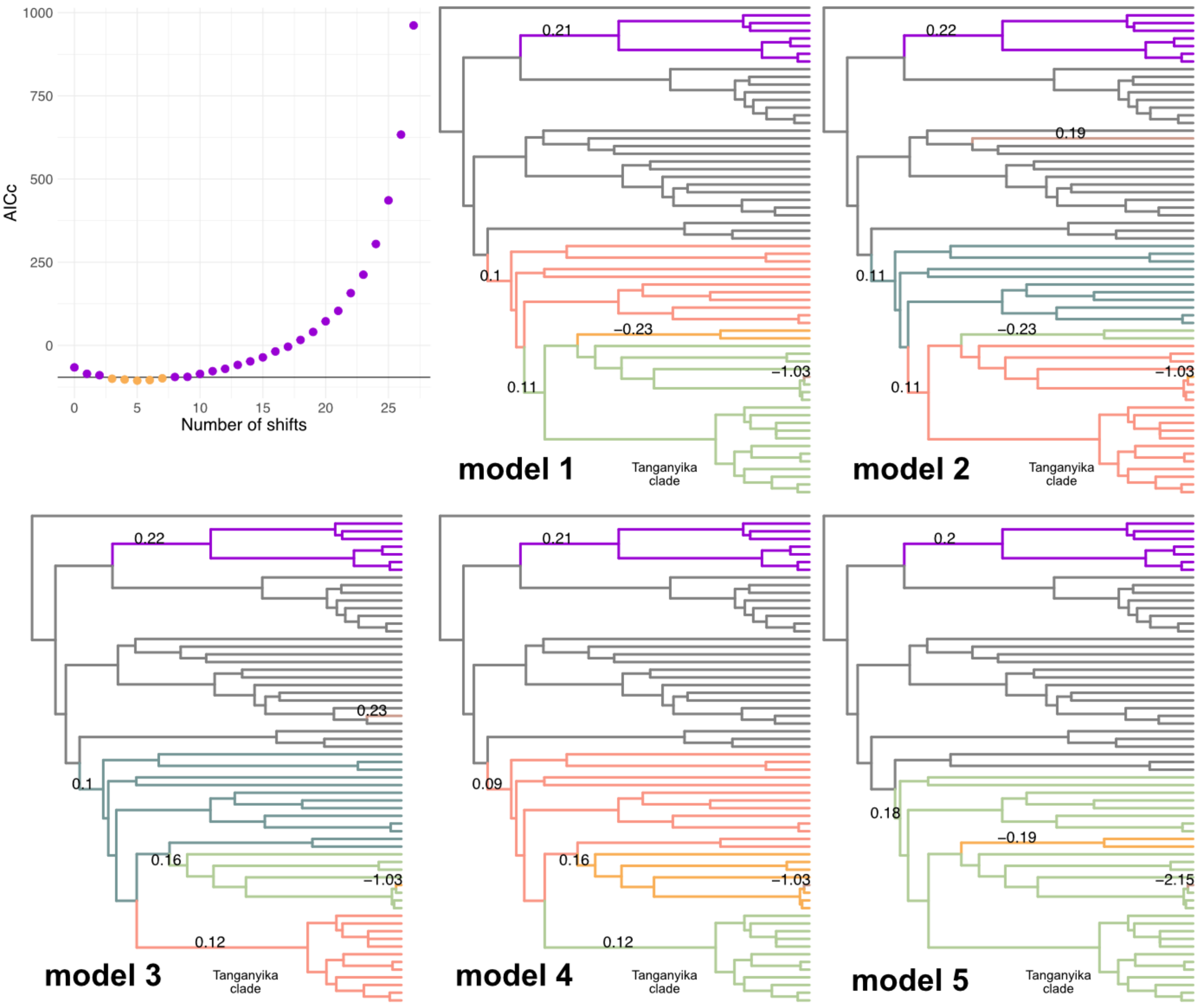

Figure S5 AICc profile and inferred Ornstein-Uhlenbeck shifts for the *l1ou* model of the sperm ratio. Shift configurations that differred by 10 AICc or less from the best configuration (model 1) are highlighted in yellow in the first plot and vizualized. Values at the shift locations indicate the magnitude and direction of the shift. sperm ratio

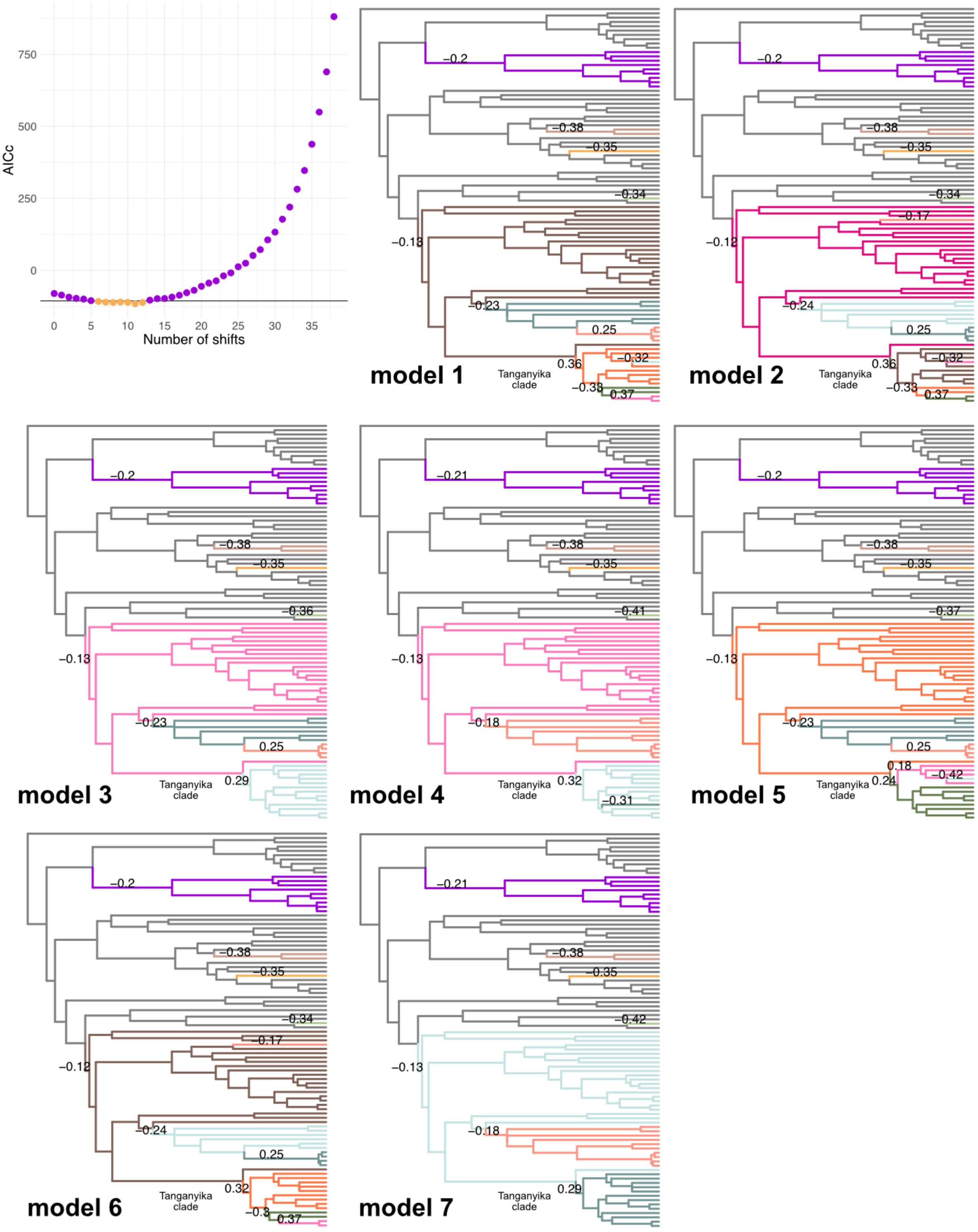

Figure S6 AICc profile and inferred Ornstein-Uhlenbeck shifts for the *l1ou* model total sperm length in cluding additional species (94). Shift configurations that differred by 10 AICc or less from the best configuration (model 1) are highlighted in yellow in the first plot and vizualized. Values at the shift locations indicate the magnitude and direction of the shift.
